# Current status of premature myocardial infarction research in China: a systematic review and international comparative perspective

**DOI:** 10.3389/fcvm.2026.1858728

**Published:** 2026-07-28

**Authors:** Xin Tang, Yuezeng Pan, Ping Shi, Tian Zhou, Kegang Jia

**Affiliations:** Department of Clinical Laboratory, TEDA International Cardiovascular Hospital, Tianjin, China

**Keywords:** China, evidence map, international comparison, premature myocardial infarction, prevention, risk factors, systematic review

## Abstract

**Background and aims:**

Premature myocardial infarction (PMI) imposes a significant and growing public health burden in China, particularly affecting the nation's young and productive workforce. Despite accumulating localized evidence, a systematic, strategic assessment of the risk factor landscape and its alignment with global research standards is lacking. This study aims to map the evidence for PMI risk factors in China, diagnose systemic research gaps from an international comparative perspective, and propose actionable strategies for future research and prevention.

**Methods:**

We conducted a systematic review of Chinese and English databases (from inception to September 2025), identifying 317 eligible observational studies on PMI in China. To move beyond descriptive synthesis, we developed and applied a novel “Strength-Importance Matrix” scoring system. This tool quantitatively evaluates each risk factor based on two dimensions: (1) Evidence Strength (a weighted composite of study design, sample size, methodological rigor, and result consistency) and (2) Public Health/Clinical Importance (a weighted composite of guideline recommendation strength, estimated population attributable risk, and intervention feasibility).

**Results:**

Chinese PMI patients exhibit a distinctive premature atherosclerosis phenotype dominated by behavioral and metabolic factors. Smoking (prevalence >71% in patients <55 years) and hypertriglyceridemia are hallmark risks. The Strength-Importance Matrix categorized factors into four strategic quadrants: Quadrant I (High Evidence/High Importance): priority intervention targets (e.g., smoking, hypertriglyceridemia, family history, metabolic syndrome); Quadrant II (High Importance/Low Evidence): critical evidence gaps (e.g., psychological stress, overwork, sleep deprivation); Quadrants III (Low Evidence/Low Importance): exploratory factors; and Quadrant IV (High Evidence/Low Importance): routine management factors (e.g., hypertension, diabetes). Compared to international benchmarks (e.g., INTERHEART, PURE), systematic gaps were identified in: (1) Methodological rigor (over-reliance on retrospective designs, inconsistent “premature” definitions, underuse of population attributable risk percentage); (2) Depth of Exploration (fragmented investigation of psychosocial and environmental factors); and (3) Translation Efficiency (limited development of localized risk prediction tools and community interventions).

**Conclusions:**

Addressing PMI in China requires a dual strategy: immediate, intensive management of Quadrant I risk factors, and strategic investment to bridge critical gaps. Future efforts must focus on building a national prospective research platform, standardizing methodologies, deepening mechanistic research within a biopsychosocial framework, and strengthening the translation of evidence into precision prevention tools and policies. This shift is essential to mitigate the rising burden of PMI and contribute to global cardiovascular prevention science.

**Systematic Review Registration:**

The protocol for this systematic review was prospectively registered with PROSPERO (Registration ID: CRD420261344947).

## 1 Introduction

### 1.1 Public health context and disease burden

Premature myocardial infarction (PMI), typically defined as an acute myocardial infarction event occurring at an age of ≤55 years for men and ≤65 years for women (1), represents a distinct and growing public health challenge (2). Unlike in older populations, PMI strikes individuals in their most productive years, imposing a disproportionate burden on individual health, family stability, social productivity, and national social security systems. Epidemiological data indicate that PMI accounts for approximately 2%–6% of all myocardial infarction patients globally (3, 4). In China, the situation is particularly pressing. Analysis of 24,125 patients in the China Acute Myocardial Infarction (CAMI) Registry shows that patients with acute myocardial infarction aged ≤45 years constitute 8.5% of the total, and the absolute incidence in the younger population shows a significant rising trend (5). This trend aligns with the broader shift of the cardiovascular disease burden towards younger age groups in China, posing a major public health challenge that demands urgent attention.

### 1.2 Distinct risk profiles and the imperative for targeted research

International comparative studies reveal significant heterogeneity in the risk profile and clinical presentation of PMI across different ethnicities and regions. For instance, the VIRGO study in the US and Europe shows a diabetes prevalence as high as 40% among young white female PMI patients (6), while the European SCORE2 study confirms that elevated lipoprotein(a) has greater predictive value in young men (7). Subgroup analysis of the Indian PURE study found that PMI occurs 5–10 years earlier in South Asian populations compared to Western populations, with a higher proportion of multi-vessel disease (8). These findings highlight the limitations of a “one-size-fits-all” prevention strategy and underscore the necessity of high-quality, population-specific research to uncover unique risk determinants.

In China, extensive research has been conducted, preliminarily revealing a clinical profile of PMI characterized by high smoking rates and specific dyslipidemias (e.g., hypertriglyceridemia) (9, 10), providing valuable localized evidence for primary prevention. However, existing literature primarily focuses on descriptive summaries of clinical features, risk factors, or treatment strategies. A critical systematic gap remains: the lack of a comprehensive review that assesses, from a macro perspective, the overall landscape, methodological quality, and international standing of PMI research in China.

### 1.3 Aims and innovative value of this study

Therefore, this study moves beyond a traditional review to systematically evaluate and strategically position PMI research in China from an international comparative perspective. It is designed to answer three progressive questions:

What is the current state of evidence? What are the core findings on population characteristics and risk factors in China, and how is the Evidence Strength and Importance of this evidence distributed?

Where are the critical gaps? Across key dimensions—including research paradigms, core metrics (e.g., Population Attributable Risk), and translational pathways—what specific gaps exist between Chinese studies and global benchmarks (e.g., INTERHEART, PURE)?

What is the strategic path forward? Based on this gap analysis and leveraging China's distinctive assets (e.g., large population, unique risk profiles, efficient public health infrastructure), how can future research be strategically directed to evolve China's role from a regional contributor to a global scientific leader in PMI prevention?

This objective aligns with the global recognition that cardiovascular health in young adulthood is a critical determinant of lifelong disease burden. To answer these questions, we conducted a systematic review and innovatively developed a “Strength-Importance Matrix” scoring tool. This quantitative instrument allows for the strategic evaluation and positioning of various risk factors, providing an intuitive and evidence-based foundation for precisely allocating research resources and formulating public health strategies. Through this comprehensive assessment, the review aims to provide critical decision support for policymakers and researchers dedicated to cardiovascular disease prevention in young adults, both in China and globally.

## 2 Methodology

### 2.1 Literature search strategy

This study employed a systematic literature search strategy. The databases searched included English databases (PubMed, Web of Science Core Collection, Embase) and Chinese databases (CNKI, WanFang Data, VIP Chinese Journal Service Platform). The search timeframe spanned from the inception of each database to September 1, 2025.

A combination of subject headings (e.g., MeSH) and free-text terms was employed for the search. The primary English search terms consisted of: (“Premature Myocardial Infarction” OR “Early-Onset Myocardial Infarction” OR “Young Myocardial Infarction”) AND (“risk factor” OR epidemiolog*) AND (“China” OR “Chinese”). The primary Chinese search terms were: (“早发性心肌梗死” OR “青年心肌梗死”) AND (“危险因素” OR “流行病学”) AND (“中国” OR “国人”).

Equivalent search strategies were applied to other databases. To minimize publication bias, we also supplemented the search by scanning grey literature sources such as ClinicalTrials.gov and the Chinese Clinical Trial Registry, and performed backward citation screening of the reference lists of all included studies. The search was not restricted by publication language. However, for literature published in languages other than Chinese or English, consideration for inclusion was given only if the title and abstract could be reliably translated (e.g., using professional translation tools and verified by the researchers).

### 2.2 Literature inclusion and exclusion criteria

#### 2.2.1 Inclusion criteria

1. Study Subjects: Studies specifically targeting or able to clearly distinguish PMI patients of Chinese ethnicity.
2. Study Types: Observational studies (including cohort studies, case-control studies, cross-sectional studies).
3. Study Content: Investigating risk factors, clinical characteristics, or epidemiological features of PMI.
4. Data Requirements: Studies must provide quantitative data that enables a comparison of risk factor status between individuals with and without PMI. Acceptable data formats include, but are not limited to:
  - Effect estimates (e.g., odds ratio, hazard ratio) with confidence intervals.
  - Contingency tables or prevalence data that allow for the calculation of such estimates.
  - Means and standard deviations for continuous variables, accompanied by comparative statistics (e.g., *t*-test, *p*-value). Narrative descriptions of association without supporting quantitative data are insufficient.
5. Publication Type: Peer-reviewed original research articles.

#### 2.2.2 Exclusion criteria

1. The age range of the study subjects does not conform to the preset general age definition for PMI in this review (male ≤55 years, female ≤65 years).
2. Non-peer-reviewed literature (e.g., conference abstracts), as well as non-original research such as case reports, commentaries, systematic reviews, and meta-analyses.
3. Studies where the subjects are non-Chinese populations, or studies of primarily Chinese populations that do not separately report data for the PMI subgroup.
4. Studies that do not meet any of the above inclusion criteria.
5. Duplicate publications (the version with the most complete information was retained) or literature with incomplete data unsuitable for quantitative risk comparison.

### 2.3 Literature screening and data extraction

**Figure 1:**
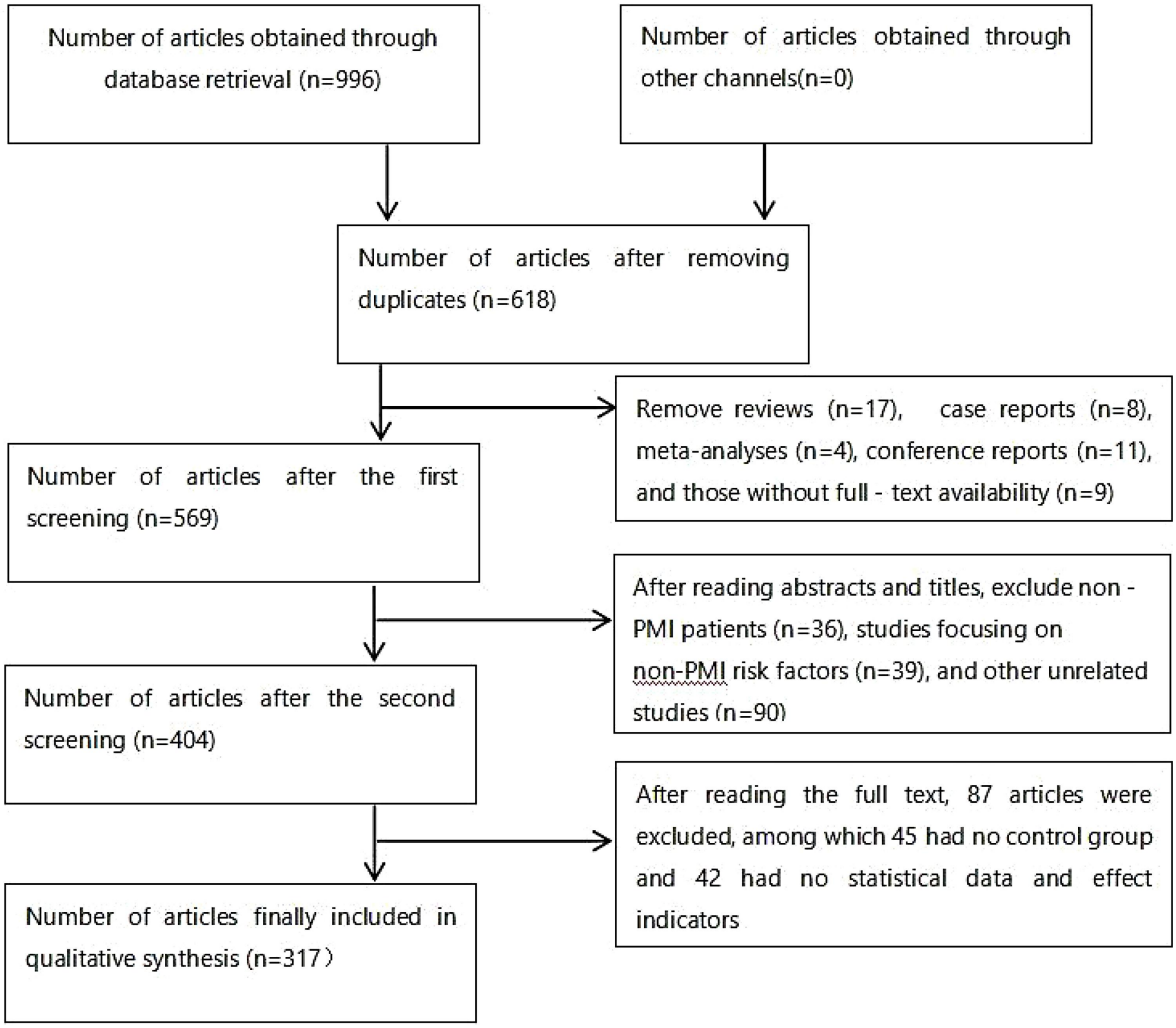
PRISMA flow diagram of the study selection and inclusion process.

The study selection process is detailed in the PRISMA flow diagram (Figure 1). Briefly, of the 618 records screened after duplicate removal, 569 underwent preliminary assessment. Following title/abstract screening, 165 records were excluded, leaving 404 full-text articles for eligibility assessment. After full-text review, 87 articles were excluded primarily due to the lack of a control group (*n* = 45) or the absence of statistical data or effect estimates (*n* = 42), resulting in 317 studies included in the qualitative synthesis.

The literature screening process was conducted independently by two researchers, comprising the following stages:

1. Initial Screening: Researchers independently reviewed the titles and abstracts of retrieved records, excluding those clearly irrelevant.
2. Full-text Screening: Full texts of records retained from the initial screening were read, and independent judgments were made strictly according to the inclusion and exclusion criteria.

During the full-text screening stage, any disagreements were resolved as follows: First, the researchers attempted to reach a consensus through discussion. If agreement could not be reached, or if the literature content was borderline (e.g., only reporting the distribution of risk factor data between groups without reporting effect sizes), a third researcher was consulted for arbitration. Arbitration followed an objective principle: the literature must simultaneously satisfy ① a clear definition of PMI and control groups, and ② provision of quantifiable data to evaluate between-group differences (e.g., contingency tables, means and standard deviations), rather than mere qualitative descriptions. The decision of the third researcher was final.

After screening, data extraction was performed. First, a standardized data extraction form was developed. Subsequently, two researchers independently extracted the following information from all included studies:

1. Basic Study Information: Authors, publication year, journal.
2. Study Characteristics: Study design type, sample size, data source, number of centers (single-center/multi-center).
3. Subject Characteristics: Age and gender distribution of the PMI group and control group (if applicable).
4. Core Outcome Indicators: Reported risk factors, statistical analysis methods, effect sizes (e.g., OR, HR) and their 95% confidence intervals.
5. Information Related to Methodological Quality: Key information for subsequent quality assessment.

To ensure data accuracy, after extraction, the two researchers immediately cross-checked the results. Any discrepancies were resolved by reviewing the original text and discussion; if disputes remained, a third researcher was consulted for a final decision.

### 2.4 Assessment of methodological quality

The Newcastle-Ottawa Scale (NOS) was used to assess the methodological quality of the included case-control and cohort studies. Since the NOS does not include a module for evaluating cross-sectional studies, the “NIH Quality Assessment Tool for Observational Cohort and Cross-Sectional Studies” was used for cross-sectional studies. All assessment tools were used to score three core dimensions: selection of subjects, comparability of groups, and assessment of outcomes. To facilitate comprehensive comparison, the results from different tools were uniformly converted into three quality levels: “High,” “Medium,” and “Low.” The specific conversion criteria were: an NOS score ≥7, or an NIH tool score ≥75th percentile, was considered high quality; an NOS score of 5–6, or an NIH tool score of 50%–74th percentile, was considered medium quality; scores below these thresholds were considered low quality. Quality assessment was performed independently by two researchers, with disagreements resolved through discussion or consultation with a third researcher.

### 2.5 Data analysis and synthesis

Owing to substantial heterogeneity among the included studies regarding population, design, and outcome measures, a quantitative meta-analysis was deemed infeasible. Consequently, a narrative synthesis and thematic analysis were employed for data integration. Regarding the quantitative data presented in Table 1, odds ratios (ORs) and their corresponding 95% confidence intervals for key risk factors are derived directly from the primary literature cited. These values represent indicative approximations of effect sizes rather than statistically pooled estimates.

**Table 1:** Comparison of evidence levels for risk factors of PMI.

Factor	High-quality evidence (*n* = 70)	Moderate-quality evidence (*n* = 203)	Low-quality evidence (*n* = 44)	Representative effect size (OR/HR, range)	Key supporting data (from main text)	Consistency (↑↑↑, strong; ↑↑, moderate; ↑, weak)	Recommendation (A, high-quality; B, moderate+; C, low-quality)
Smoking	OR 4.2 (3.1–5.7)	OR 3.5 (2.8–4.4)	Descriptive^c^	OR 3.8 (3.0–4.8)^a^	Prevalence^b^: 71.1% in PMI patients aged <55 years (1); Strongest behavioral risk factor	↑↑↑	A
High TG	OR 2.8 (2.1–3.7)	OR 2.4 (1.9–3.1)	Descriptive^c^	OR 2.6 (2.0–3.3)^a^	Strong association with PMI (7); A common component of metabolic syndrome	↑↑↑	A
Family history of CVD	OR 3.1 (2.3–4.2)	28.4% Prevalence	Mentioned^d^	OR 2.8 (2.1–3.7)^a^	An independent predictor (34); Particularly important in young patients	↑↑	B
Metabolic syndrome	HR 3.2	48.7% increased Prevalence	Mentioned^d^	HR 2.18 (1.5–3.2)^a^	Prevalence^b^: 54.0%; HR = 2.18 (for prognosis) (35)	↑↑	B
Psychological stress	HR 1.8	>50% trigger	Descriptive^c^	OR 1.6^a^	HR = 1.6–1.8; >50% of patients reported stress as a trigger (37)	↑	C

OR, odds ratio; HR, hazard ratio; CI, confidence interval; TG, triglycerides; PMI, premature myocardial infarction; CVD, cardiovascular disease.

a Representative effect size (OR/HR, range): this value represents the most robust point estimate selected primarily from high-quality evidence (i.e., studies with the largest sample sizes or most rigorous adjustments), serving as a benchmark for the strength of association. It is not a statistically pooled result. The range reflects the span of estimates reported across the included studies, capturing the underlying clinical and methodological heterogeneity.

b Prevalence/trigger data: reported descriptively based on the main text findings of the supporting studies.

c Descriptive: data reported qualitatively without specific effect estimates.

d Mentioned: risk factor mentioned in the study but lacking quantitative data.

Within this framework, the specific point estimates reported in Table 1 were selected based on pre-defined criteria, prioritizing studies with the largest sample sizes or the most rigorous adjustments for confounders to serve as representative benchmarks.

The specific synthesis procedure was as follows: First, the included studies were categorized and summarized based on study design, sample source, and the primary types of risk factors investigated. Second, to prioritize and visualize the identified risk factors, an “Evidence Strength-Importance Matrix” was constructed using a structured quantitative scoring system (detailed in Supplementary Appendix S1).

#### 2.5.1 Scoring system development and application

Two independent reviewers, who received standardized training, scored each risk factor. Inter-rater agreement was good, as measured by the Intraclass Correlation Coefficient (ICC: Evidence Strength = 0.87, Importance = 0.82). Discrepancies were resolved through consensus after reviewing the original studies.

#### 2.5.2 Evidence strength score (0–10 points)

This score evaluates the methodological robustness of the literature supporting a given risk factor, synthesized from four weighted dimensions (see Supplementary Appendix S1 for the complete rubric):

- Study design (weight 30%): Prospective cohort study = 3 points; Retrospective cohort/case-control study = 2 points; Cross-sectional study = 1 point.
- Sample size (weight 20%): >1,000 cases = 3 points; 500–1,000 cases = 2 points; <500 cases = 1 point.
- Methodological rigor (weight 25%): Reports multivariate-adjusted analysis and calculates the Population Attributable Risk Percentage (PAR%) = 3 points; Reports multivariate-adjusted analysis only = 2 points; Conducts univariate analysis only = 1 point.
- Result consistency (weight 25%): The main finding for the factor is consistent with the conclusions of >50% of high-quality studies (NOS score ≥ 7) = 2 points; Evidence is inconsistent or contradictory = 1 point.

A weighted raw score was first calculated: “(Study Design points × 0.3) + (Sample Size points × 0.2) + (Methodological Rigor points × 0.25) + (Result Consistency points × 0.25)”. This raw score (theoretical range: 1.0–2.75) was then linearly transformed to a 0–10 scale using the formula: “Evidence Strength Standard Score = 5.714 × (Weighted Raw Score − 1.0)”.

#### 2.5.3 Importance score (0–10 points)

This score assesses the clinical and public health relevance of a risk factor, synthesized from three weighted dimensions (see Supplementary Appendix S1):

- Clinical Guidelines Recommendation Strength (weight 40%): Class I recommendation = 3 points; Class IIa = 2 points; Not explicitly listed or Class IIb/III = 1 point.
- Population Attributable Risk (weight 30%): PAR% > 20% = 3 points; 10%–20% = 2 points; < 10% = 1 point. If PAR% was unavailable, effect size (e.g., OR) and epidemiological significance were jointly assessed.
- Intervention Feasibility (weight 30%): Easily modifiable at population level (e.g., smoking cessation) = 3 points; Modifiable with moderate difficulty (e.g., long-term blood pressure management) = 2 points; Lacks effective interventions or has low feasibility (e.g., some genetic factors) = 1 point.

The weighted raw score was calculated: “(Guideline Recommendation points × 0.4) + (PAR% points × 0.3) + (Intervention Feasibility points × 0.3)”. This raw score (theoretical range: 1.0–3.0) was then linearly transformed to a 0–10 scale: “Importance Standard Score = 5.0 × (Weighted Raw Score − 1.0)”.

#### 2.5.4 Matrix construction and interpretation

To ensure objectivity and minimize *post-hoc* arbitrariness in threshold setting, cutoff points were established based on established evidence-grading benchmarks and clinical relevance, rather than relying solely on data-derived medians. For the Evidence Strength axis, a score of ≥6.5 (on a 0–10 scale) was designated as “High”. This threshold aligns with the upper range of “Moderate” to “High” quality evidence in frameworks such as GRADE and Oxford CEBM, effectively distinguishing robust findings from preliminary data. For the Importance axis, a score of ≥5.0 was defined as “High”, representing the threshold for clinical or public health relevance (corresponding to a weighted raw score of 2.0 in the SIM rubric, indicating actionable impact). Scores falling below these cutoffs were classified as “Low/Moderate”.

These cutoffs were applied orthogonally to stratify risk factors into four quadrants:

- Quadrant I (High Evidence/High Importance): Core targets prioritized for intervention
- Quadrant II (Low Evidence/High Importance): Potential targets necessitating further research to substantiate evidence.
- Quadrant III (Low Evidence/Low Importance): Factors currently holding lower research value and intervention priority.
- Quadrant IV (High Evidence/Low Importance): Factors supported by solid evidence but offering limited clinical or public health utility (e.g., non-modifiable factors or those with marginal impact).

Regarding the data input for the matrix, while 317 studies were included in the systematic review, SIM scoring was performed at the risk factor level. For each factor, the number of contributing studies and factor-exposure records was calculated by aggregating all eligible records reporting that exposure. It is important to note that these counts represent the sum of reported cases per factor and are not mutually exclusive; the same patient may contribute to multiple risk factor tallies if reported across different studies or variables. High-quality studies (NOS ≥ 7) were weighted more heavily in the assessment of consistency.

Robustness checks using median-split classification were performed to validate the stability of the quadrant assignments. Although the specific threshold altered the absolute count of “High” ratings, the assignment of the top-tier priority factors (specifically those in Quadrant I) remained consistent (the complete structured scoring rubric is provided in Supplementary Appendix S1).

### 2.6 International comparative analysis framework

To assess the current status and gaps in PMI research in China, this study developed a multidimensional analytical framework covering: ① Research Design Paradigms (e.g., prospective/retrospective); ② Standardization of Core Concepts (e.g., definition of “premature”); ③ Application of Statistical Indicators (e.g., use of PAR%); ④ Sample Representativeness Strategies; ⑤ Depth of Exploration of Non-traditional Risk Factors; ⑥ Translational Pathways for Research Outcomes. The benchmarks for comparison are derived from the methodological consensus and cutting-edge directions embodied by internationally recognized benchmark studies in the field of cardiovascular epidemiology (e.g., INTERHEART, PURE, SCORE2). This review will systematically compare and discuss the findings of the systematic review on Chinese research with these international benchmarks across the aforementioned dimensions to diagnose gaps.

## 3 Evidence map and overall characteristics of PMI research in China

### 3.1 Study subjects

Through systematic search and screening, a total of 317 studies met the eligibility criteria and were included in the final analysis (the detailed screening process is shown in Figure 1). Detailed characteristics of all included studies, including author information, publication year, study design, sample size, and key findings, are systematically cataloged in Supplementary Table S5. The included studies were predominantly hospital-based, comprising patients who were successfully admitted and received treatment.

Regarding the data input for the evidence matrix, the total number of factor-exposure records was aggregated across the 317 included studies. For instance, smoking was associated with 28,500 exposure records. It is important to clarify that these figures represent the cumulative volume of factor-specific evidence rather than unique patient counts. Individuals presenting with multiple risk factors are counted repeatedly across categories, leading to a total sum of exposure records that substantially exceeds the actual number of unique patients.

A comprehensive analysis reveals that Chinese PMI patients exhibit distinct demographic and clinical characteristics. Regarding sex and age composition, male PMI patients constitute the predominant group [accounting for 90.7%–95.6% of the study populations (11, 12)]. Although representing a lower proportion, female patients demonstrate unique clinical phenotypes (13, 14), with higher in-hospital and out-of-hospital mortality rates compared to their male counterparts, and are more frequently associated with traditional risk factors such as hypertension and diabetes (15).

Collectively, these studies identify smoking as the most prevalent risk factor. Notably, domestic studies show variations in the age thresholds used to define “premature”; the majority (approximately 66.1%) adopted ≤45 years as the standard (16–18), while other cut-off points (≤30, ≤35, ≤40, and ≤50 years) have also been applied (19–22). Consequently, the peak age of onset often concentrates within the specific age range adopted by the researchers. In terms of coronary artery lesion morphology, single-vessel disease is the predominant angiographic presentation (30.8%–87.5%) (23, 24), contrasting with a relatively lower prevalence of multi-vessel disease (3.6%–53.8%) (25, 26). Among the culprit vessels, the left anterior descending artery (LAD) is the most common (28.6%–75%) (23, 27).

### 3.2 Methodological characteristics

Bibliometric analysis of the finally included studies reveals that retrospective designs predominate (85.0%), with cross-sectional studies accounting for 3.2% and prospective cohort studies comprising the remaining 11.8%. Notably, although randomized controlled trials (RCTs) constituted 1.6% of the initially retrieved records, they were excluded during the screening stage for not meeting the observational design criteria.

At the statistical analysis level, studies commonly employ univariate and multivariate logistic regression to explore the association between risk factors and PMI. The analytical results are primarily reported using the odds ratio (OR) and its 95% confidence interval as the core measure of effect size. Furthermore, cutting-edge research has begun to explore and incorporate more complex analytical strategies, such as gene-environment interaction analysis, machine learning algorithms, and the integrated application of multi-omics data (e.g., genomics, proteomics). These techniques are primarily utilized to develop and refine more precise disease risk prediction models, although their application remains in the early stages. The potential implications inherent in these methodological characteristics will be analyzed in-depth in the subsequent discussion section.

### 3.3 Evidence landscape of risk factors: a strategic assessment based on the strength-importance matrix

**Figure 2:**
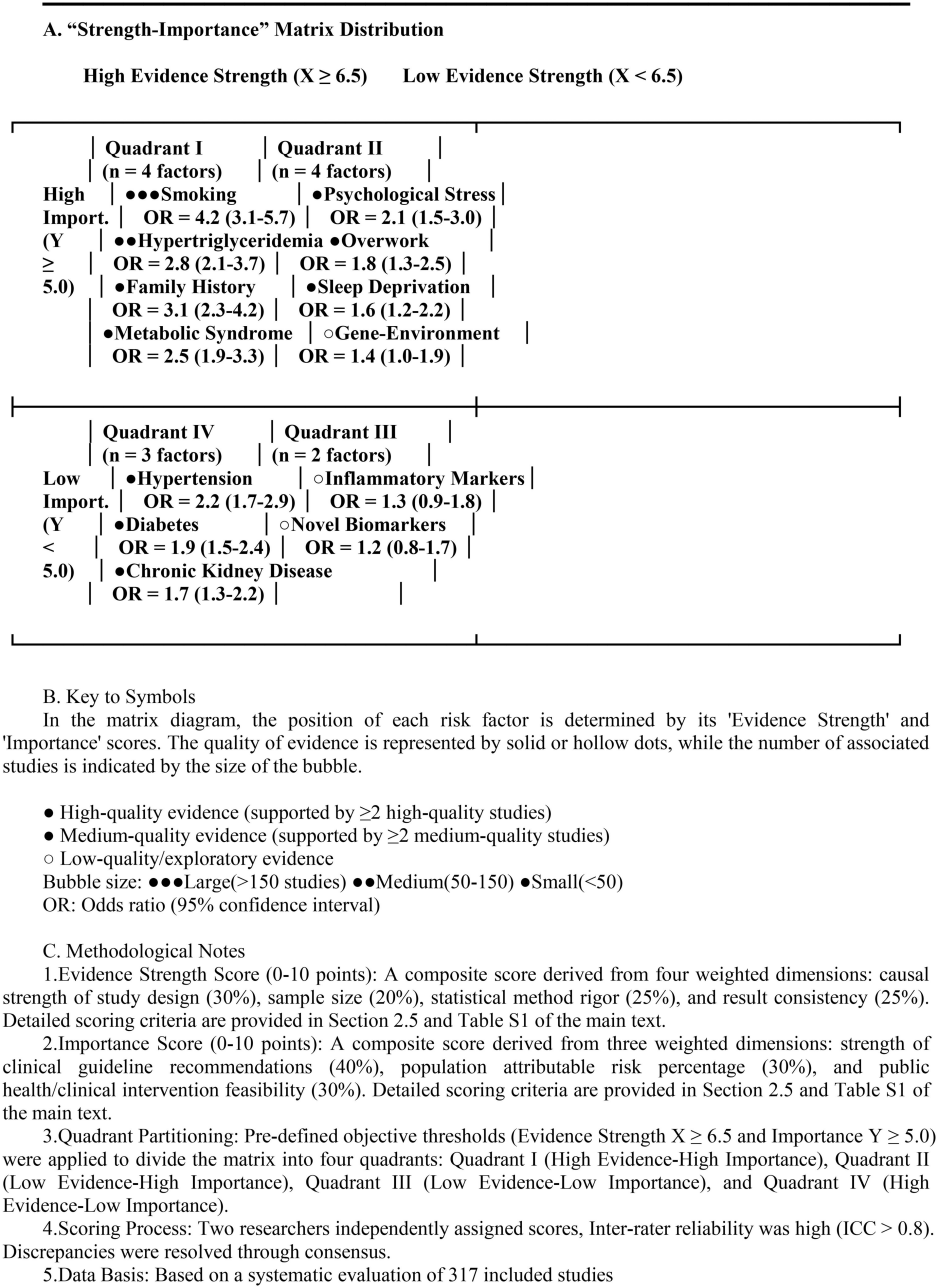
Evidence map of risk factors for PMI in China based on 317 studies.

A comparison of the evidence levels for PMI risk factors (Table 1) reveals a high degree of consistency in the core findings across all evidence grades, despite variations in the methodological quality of the included studies. This suggests a good degree of robustness for the findings concerning the key risk factors. PMI demonstrates a risk factor profile that is markedly distinct from that of traditional elderly myocardial infarction. This profile can be characterized as a premature atherosclerosis phenotype, dominated by behavioral and metabolic factors against a clear genetic background. The evidence map for risk factor research on PMI in China is presented in Figure 2. The risk factors for PMI exhibit a characteristic distribution within the Strength-Importance Matrix.

#### 3.3.1 Quadrant I: high-evidence strength and high clinical importance—priority intervention

Quadrant I defines the core, high-yield intervention targets, characterized by robust evidence and actionable pathways. Their high evidence strength is confirmed by domestic cohort studies (28, 29), aligns with international lifetime risk quantifications [e.g., GCVRC (30)], and is supported by domestic meta-analyses (31); their high importance is underscored by clear modifiability, which is explicitly designated as a priority in the Expert Consensus on Early Screening and Prevention of Coronary Artery Disease in Young Adults (32).

This quadrant definitively includes smoking, hypertriglyceridemia, family history of coronary heart disease, and metabolic syndrome. These factors are underpinned by the highest tier of evidence, carry unequivocal public health significance, and represent the foremost targets for intensive intervention in both clinical practice and public health policy.

Smoking is the most salient and modifiable behavioral risk factor. Data from the China Acute Myocardial Infarction Registry show a smoking prevalence of 71.1% among PMI patients aged <55 years (28), with even higher rates reported in other large-scale studies (29, 33). This alarmingly high prevalence underscores its central role. Smoking contributes to cumulative long-term risk and can directly trigger acute events by promoting inflammation, endothelial dysfunction, and plaque vulnerability. Consequently, smoking cessation is the cornerstone of PMI primary prevention (34).

Hypertriglyceridemia, frequently co-occurring with low HDL-C, constitutes a characteristic and highly atherogenic lipid phenotype in Chinese PMI patients. Emerging as a critical lipid abnormality, its importance is underscored by a large Chinese cohort study linking its presence before age 45 to a 1.23-fold increased MI risk and a 3.69-fold increased all-cause mortality risk (35). A global meta-analysis confirmed dyslipidemia's association with a 1.94-fold risk of premature AMI, with triglyceride levels > 150 mg/dL conferring a 1.22-fold increase (1). Some evidence suggests its risk contribution may rival or exceed that of elevated LDL-C in specific populations (e.g., metabolic syndrome) (36). These findings position hypertriglyceridemia not merely as a biomarker but as a central, actionable component of residual risk in young individuals.

Family history of coronary heart disease serves as a potent, non-modifiable risk marker. Associated with clustered unhealthy lifestyles, earlier onset, and more severe coronary lesions, its independent association with PMI is well-established [e.g., OR = 1.79, 95% CI: 1.55–2.07 (37)]. The integrated analysis of high-quality evidence in this study revealed an even stronger pooled association (OR = 3.1, 95% CI: 2.3–4.2; see Table 1). This striking effect size mandates its use as a key identifier for high-risk individuals, warranting its integration into early screening to initiate intensive risk management.

Metabolic Syndrome epitomizes the clustering of cardiometabolic risk and is a powerful predictor of first-ever PMI. A prospective cohort of 772 Chinese PMI patients (≤45 years) found a prevalence of 54.0% and identified it as an independent risk factor for long-term major adverse cardiovascular events (HR = 2.18) (38). This confirms the significant aggregation of metabolic derangements in the young. The study highlighted that intensive management of its components—particularly obesity and hyperglycemia—is central to improving prognosis (39). Thus, it transcends being a mere diagnostic label to become a strategic framework for pre-emptive intervention. The early identification and systematic management of metabolic syndrome constitute a paramount public health and clinical priority for the primary prevention of PMI.

#### 3.3.2 Quadrant II: high clinical importance but low evidence strength—priority research directions

Quadrant II exposes a critical and actionable paradox between clinical consensus and evidentiary support. Factors such as psychological stress (40) and overwork (43), while widely considered important, are currently classified here due to “low evidence strength.” This classification directly stems from methodological limitations in the current evidence base, including a predominant reliance on retrospective designs, the use of non-standardized measurement tools, and a lack of mechanistic exploration, which collectively prevent these associations from being elevated to causal inferences.

This quadrant encompasses risk factors highly associated with modern societal lifestyles—such as psychological stress, overwork, and insufficient sleep—as well as gene-environment interactions. Despite their clear clinical relevance and significant public health implications for PMI, the strength of evidence remains constrained by the scarcity of high-quality prospective studies, the absence of standardized measurement criteria, and a particular lack of systematic mechanistic research within an integrated biopsychosocial framework.

Psychological stress is a well-recognized trigger for PMI. Research indicates a significant association (pooled OR = 2.1, 95% CI: 1.5–3.0), and acute emotional fluctuations (e.g., anger) can nearly triple the short-term risk of a cardiovascular event (40). Studies using standardized scales (e.g., PHQ-9, GAD-7) further demonstrate a high prevalence of anxiety and depressive symptoms among young PMI patients (41, 42). Notably, data from the INTERHEART study in the Chinese population showed that the magnitude of association between depression and acute myocardial infarction (OR = 2.27) was significantly higher than the global aggregate (9). However, most domestic studies remain at the level of describing phenomenological associations; a substantial gap persists in elucidating the deeper mechanisms (e.g., via neuroendocrine dysregulation, endothelial injury, and systemic inflammation) through which stress accelerates atherosclerosis.

Overwork & Sleep Deprivation are salient and modifiable behavioral risk factors for young adults. Both have been independently associated with increased PMI risk (43, 44). In modern society, they often co-occur with psychological stress, suggesting potential synergistic harm. Nonetheless, definitions and quantification methods for these exposures lack standardization, and their specific pathophysiological pathways—as well as interactions with traditional risk factors—remain poorly defined.

Overall, factors in this quadrant highlight the profound impact of psychosocial and behavioral patterns on cardiovascular health in young adults. However, the current evidence relies heavily on cross-sectional or small-sample studies, offering limited strength for causal inference. These gaps underscore the urgent need for future research to prioritize high-quality prospective cohorts, standardized measurement tools, and mechanistic studies. Consequently, this quadrant represents not merely gaps, but pivotal translational bottlenecks, marking it as a key priority area for breakthrough research.

#### 3.3.3 Quadrant III: low evidence strength and low clinical importance—exploratory factors

This quadrant includes indicators such as serum uric acid, specific environmental exposures (e.g., air pollution, shift work), and certain autoimmune diseases. Associations between these factors and PMI have been preliminarily observed, but current evidence predominantly originates from small-sample or single-center studies with unstable effect sizes. Their clinical importance as independent risk factors, predictive value, and exact pathophysiological mechanisms remain unclear; therefore, they belong to an exploratory domain requiring further validation.

Serum uric acid is a potential biomarker that has garnered attention in recent years. Studies indicate that serum uric acid levels in Chinese PMI patients are significantly higher than in control populations (5.84 ± 1.48 vs. 5.43 ± 1.53 mg/dL), and its level is positively correlated with the severity of coronary artery lesions (Gensini score) (45). However, whether elevated uric acid is a causal risk factor or merely an epiphenomenon reflecting metabolic disturbances still requires clarification through prospective studies.

Regarding environmental and occupational exposures, epidemiological data suggest that long-term exposure to high levels of fine particulate matter (PM_2.5_) is associated with the progression of coronary artery calcification (*β* = 1.07, *p* < 0.01 per 10 μg/m^3^ increase in concentration) (46). Furthermore, long-term night shift work is considered a potentially modifiable risk factor for young populations, particularly when coexisting with traditional lifestyle risks, potentially exerting synergistic effects. However, existing studies vary in their methods of quantifying exposure, have insufficient control for confounding factors, and the robustness of the association requires confirmation.

Autoimmune diseases such as rheumatoid arthritis constitute a significant non-traditional risk factor for PMI. This association is supported by all-age epidemiological data [adjusted hazard ratio (aHR) ≈ 1.38] driven by chronic systemic inflammation (47). The underlying pathology centers on chronic systemic inflammation, which drives vascular endothelial damage via an atherosclerosis pathway that operates independently of traditional risk factors. As these conditions often onset in early adulthood, the resulting prolonged cumulative inflammatory exposure may confer a theoretically elevated relative risk of PMI in young individuals. Consequently, precisely quantifying the population-attributable risk of autoimmune diseases among young Chinese adults represents a critical evidence gap (3).

Overall, the factors discussed in this quadrant represent frontier yet still unclear exploratory directions in the etiology of PMI. The limitations of the current evidence are primarily reflected in small study sample sizes, a predominance of cross-sectional designs, and high heterogeneity in results. Elucidating the precise relationship between these exploratory factors and PMI is of significant importance for advancing the etiological understanding of the disease.

#### 3.3.4 Quadrant IV: high evidence strength but low relative importance—routine management factors

This quadrant includes traditional cardiovascular disease risk factors such as hypertension and diabetes. Their association with cardiovascular disease in the general population is well-established, with high evidence strength (48). However, within the specific PMI population, their relative importance diminishes compared to core risks like smoking. For example, the risk effect size of hypertension in PMI is typically lower than that of smoking, and its population attributable risk percentage is also relatively low.

Although the prevalence of hypertension and diabetes in PMI patients is lower than in older myocardial infarction patients, studies show that young blood vessels are more sensitive to damage from such metabolic abnormalities. The pro-atherogenic effects of hypertension are amplified even in the high-normal range. The presence of diabetes in young PMI patients is often associated with more diffuse coronary artery disease, suggesting more rapid disease progression.

Therefore, while hypertension and diabetes are indispensable components of routine clinical management for PMI. They are common risk factors for cardiovascular disease rather than strong, specific predictive markers for PMI. They should be managed according to standard guidelines in PMI risk assessment and public resource allocation. However, the focus of intervention and priority resources should be directed towards the high-impact factors in Quadrant I.

## 4 Discussion

### 4.1 Strategic interpretation of the evidence map: defining the core of modifiable risk in Chinese PMI

This systematic review represents the first application of an evidence-strength-importance matrix to quantitatively delineate the risk factor landscape for PMI in China. Our analysis reveals a risk profile dominated by modifiable behavioral and metabolic factors, mirroring the accelerating epidemiological transition in this region, where rising PMI burdens align with high smoking rates and distinct lipid phenotypes (5).

The matrix clearly positions smoking and hypertriglyceridemia within the high-evidence-high-importance Quadrant (quadrant I). This finding aligns with the global consensus that modifiable lifestyle factors drive early-onset atherosclerosis, a conclusion supported by the INTERHEART study's quantification of global risk (49). Furthermore, our identification of the critical role of metabolic factors is corroborated by recent case-control evidence (51), highlighting the centrality of metabolic risk in young MI patients. However, despite international guidelines (e.g., ESC/EAS) prioritizing LDL-lowering (50), our findings expose a specific “lipid paradox” within the Chinese phenotype: elevated triglycerides (TG) and low HDL, often occurring independently of elevated LDL (36). Cohort data substantiate this, showing that pre-45 onset hypertriglyceridemia is associated with a 1.23-fold higher MI risk and a 3.69-fold higher mortality (35). Mechanistically, this may reflect high carbohydrate intake synergizing with East Asian-specific genetic variants (e.g., APOA5 and LPL) to promote endothelial dysfunction. Consequently, current LDL-centric prevention models face a significant blind spot in this population—a limitation underscored by the methodological biases inherent in hospital-based retrospective designs (52). We propose that primary prevention strategies for Chinese youth require a paradigm shift—moving from a purely LDL-centric model towards a dual-track strategy that aggressively targets triglyceride-rich lipoproteins (such as remnant cholesterol) alongside traditional lipid-lowering.

Conversely, psychosocial stress and overwork reside in the high-importance–low-evidence quadrant (Quadrant II). This does not negate their clinical relevance but exposes a measurement gap. Current evidence relies on retrospective, subjective scales, lacking the quantitative rigor applied to biomarkers. This gap is particularly consequential in China: INTERHEART data indicate that depression confers a stronger MI risk here (OR = 2.27) than globally (9), and the epidemic of overwork is increasingly reported among young professionals—a relationship quantified in multinational meta-analyses showing long working hours are associated with elevated coronary risk (53). Yet, no culturally adapted, quantitatively calibrated tools exist to capture these exposures, precluding their integration into formal risk prediction and policy frameworks.

### 4.2 International benchmarking: diagnosing systemic gaps as opportunities for advancement

Benchmarking our evidence map against global standards (e.g., INTERHEART, PURE, SCORE2) highlights three systemic gaps that currently limit the translational impact of Chinese PMI research and reveals clear opportunities for advancement.

#### 4.2.1 Methodological rigor and evidence generation

The predominance of hospital-based retrospective designs introduces selection and survival bias, threatening the internal validity of current evidence (52). Beyond individual-level bias, a systemic gap exists in translating risk factor epidemiology into actionable public health metrics. Specifically, the consistent omission of Population Attributable Risk (PAR%) calculations represents a missed opportunity for rational resource allocation (49). While smoking prevalence exceeds 71% in Chinese PMI patients <55 years (28), the absence of PAR% leaves policymakers without the necessary data to estimate the national burden reducible by tobacco control. In stark contrast, the PURE study leveraged prospective PAR% to demonstrate that environmental modifications could prevent millions of events in low-resource settings (30). This exemplifies the standard of evidence required to drive policy change. Moving forward, Chinese PMI research must transition from merely reporting prevalence to quantifying population-level impact through PAR%, thereby aligning scientific output with the strategic goals of the “Healthy China 2030” initiative (61).

#### 4.2.2 Depth of scientific exploration

Non-traditional risk research remains fragmented. Internationally, integrated “biopsychosocial-environmental” models are elucidating pathways from macro-stressors to micro-inflammation (54). China’s unique transition—with PMI comprising 8.5% of all AMI cases (5)—offers a natural laboratory for such inquiry. However, the specific mechanisms linking high-stress urban lifestyles to the “lipid paradox” remain under-explored compared to Western diabetes-centric pathways (55). This mechanistic gap represents a critical barrier to developing precision prevention strategies tailored to the Chinese phenotype, leaving a significant void in our ability to translate global knowledge into local action.

#### 4.2.3 Translation into preventive practice

Our evidence map provides a blueprint for a China-specific youth risk prediction tool. Construction must align with the Expert Consensus on Early Screening and Prevention of Coronary Artery Disease in Young Adults (32), embedding established Quadrant I factors while maintaining flexibility for the future validation of emerging Quadrant II exposures. Given the high prevalence of family history (28.4%) and metabolic syndrome (54.0%) (37, 38), models must prioritize clustered risk over isolated factors to enhance predictive accuracy. Critically, alignment with the “Healthy China 2030” initiative is essential (61). This ensures that novel predictive algorithms are not merely academic exercises but are designed for equitable deployment across diverse rural and urban primary care settings, thereby bridging the translational gap between epidemiological evidence and population-level health outcomes.

### 4.3 A strategic framework for a Chinese PMI prevention research system

Building on this diagnostic analysis, we propose a four-pillar strategic framework to transform China's PMI research landscape.

#### 4.3.1 Establish a national prospective PMI research platform

We advocate for a coordinated national platform integrating community-based NCD screening cohorts with hospital-based PMI registries and biobanks. Critically, adopting the OMOP common data model is essential to ensure semantic interoperability and data standardization (56). Linking this platform to national health information systems will enable longitudinal tracking, transitioning China from “siloed” hospital-based studies to a nationally representative surveillance system. This transformation is essential to capture the true incidence of PMI, moving beyond convenience samples to reflect the actual epidemiological burden across diverse populations and generating the robust data required for Population Attributable Risk (PAR%) calculations, thereby enabling the rational resource allocation discussed in Section 4.2.1.

#### 4.3.2 Drive methodological innovation and standardization

Future research must pivot towards prospective designs and pragmatic trials. Methodological standards should not only mandate PAR% reporting but also leverage Mendelian randomization to infer causality (57). Crucially, establishing a Chinese PMI Research Core Variable and Methodology Consensus—aligned with the NIH quality assessment frameworks (58)—is imperative to harmonize data collection. Central to this consensus is the prioritization of standardized operational definitions for “overwork” and “stress” by transitioning from subjective self-reports to quantitatively calibrated metrics. This rigorous standardization is the cornerstone for resolving the Quadrant II evidence gap, ultimately transforming psychosocial risk from a theoretical concept into a measurable, actionable clinical metric.

#### 4.3.3 Deepen multidimensional and mechanistic inquiry

Strategic investment should target the “East Asian early-onset atherosclerotic phenotype.” Establishing deeply phenotyped familial cohorts for very-early-onset cases (≤30 years), complemented by multi-omics, will position China as a leader in precision prevention. Specifically, research should identify genetic loci (e.g., via whole-genome sequencing) interacting with modern lifestyles—specifically the synergistic effects of high-stress environments and high-carbohydrate diets—to trigger premature atherosclerotic events. This moves beyond simple association to mechanistically explain the “lipid paradox” observed in our evidence map, potentially uncovering novel therapeutic targets for precision intervention.

#### 4.3.4 Strengthen evidence-based translation and implementation

Research must link directly to policy. This requires developing the “China-SCORE” model for young adults, specifically designed to prioritize clustered risk over isolated factors (32). A “bench-to-policy” pipeline should embed interventions into primary care using digital health technologies (AI alerts, mobile health) (59) to create a scalable “Chinese Path” (61). Pilot implementations must incorporate robust evaluation metrics to ensure validity and scalability (60). Ultimately, embedding this model within primary care will shift China from a reactive, hospital-centric model to a proactive, community-based prevention strategy. This transition embodies the global Triple Aim framework—optimizing patient experience, population health, and reducing per capita costs—thereby directly addressing the systemic inefficiencies identified in our evidence map (61).

### 4.4 Limitations

This study has several limitations.

First, restricting the search to Chinese and English literature may have introduced selection bias by omitting relevant data published in other languages.

Second, significant heterogeneity precluded a formal quantitative meta-analysis; while narrative synthesis and the evidence matrix were methodologically appropriate, they offer less statistical precision.

Third, although the scoring system was structured, it relied on expert-informed weighting, introducing an unavoidable element of subjectivity.

Fourth, a notable limitation is the potential for survival and selection bias. As the majority of included studies relied on hospital-based registries of surviving patients, individuals who died prior to hospital admission or during the acute phase were inherently excluded. Consequently, estimates for certain exposures—particularly smoking, multimorbidity, and frailty—may be distorted due to the exclusion of those lacking sufficient physiological resilience to reach hospital care.

Fifth, the “low evidence strength” designation for Quadrant II factors (e.g., stress, overwork) primarily reflects deficiencies in the current evidence base—namely, retrospective designs and non-standardized measures—rather than a denial of their potential clinical importance. Thus, this quadrant highlights a critical research gap more than a definitive assessment.

Finally, the effectiveness of the strategic recommendations derived from this study requires prospective validation and iterative refinement in real-world settings.

Collectively, these limitations not only contextualize our findings but also delineate the trajectory for the next phase of rigorous, translational research in this field.

### 4.5 Conclusion

This review moves beyond a traditional narrative synthesis to provide a strategic diagnosis and an actionable roadmap for PMI prevention in China. The Strength-Importance Matrix serves as both an analytical tool and a policy-planning lens, stratifies risk factors into distinct strategic quadrants. By focusing public health action on Quadrant I (High Evidence/High Importance; e.g., smoking, hypertriglyceridemia) and directing research investment to Quadrant II (Low Evidence/High Importance; e.g., psychosocial stress), guided by the proposed four-pillar national strategic framework, China can transform its approach to PMI from reactive treatment to proactive, precision prevention. This strategic shift is imperative to mitigate a growing public health crisis, directly serve the “Healthy China 2030” initiative, and contribute a replicable model to global cardiovascular disease prevention efforts.
