# Supplementary material for: Current status of premature myocardial infarction research in China: a systematic review and international comparative perspective
Source: Front Cardiovasc Med. 2026 Jul 28;13:1858728. doi: 10.3389/fcvm.2026.1858728 (PMC13457063; doi:10.3389/fcvm.2026.1858728)
Supplement: Supplementary file 1 [file Datasheet1.pdf]

Appendix S1: Structured Scoring Rubric for the Strength-Importance Matrix (SIM)

This appendix details the scoring system developed to quantify the **Evidence Strength** and **Clinical/Public Health Importance** of each risk factor for the **Strength-Importance Matrix (SIM)**. Two independent reviewers assigned scores based on the predefined criteria below. Discrepancies were resolved through discussion. The final scores (0-10) were obtained by applying a linear transformation to the weighted raw scores, standardizing them to a common scale for comparative visualization and strategic analysis.

Table S1: Evidence Strength Scoring Rubric (0-10 points)

This score evaluates the methodological robustness of the scientific literature supporting a given risk factor.

Calculation:

Calculation: Weighted Raw Score = (Study Design Score × 0.30) + (Sample Size Score × 0.20) + (Methodological Rigor Score × 0.25) + (Result Consistency Score × 0.25)

Final Standard Score (0-10)= 5.714 × (Weighted Raw Score - 1.0)

The multiplier 5.714 linearly scales the score, mapping a theoretical raw score range of [1.0, 2.75] to the standard range of [0, 10]

Dimension	Weight	Scoring Criteria (Original Points)
1. Study Design	30%	**3 Points**: Prospective Cohort Study<br>**2 Points**: Retrospective Cohort or Case-Control Study<br>**1 Point**: Cross-sectional Study
2. Sample Size (Number of PMI cases)	20%	**3 Points**: > 1000 cases<br>**2 Points**: 500 – 1000 cases<br>**1 Point**: < 500 cases
3. Methodological Rigor	25%	**3 Points**: Reports **multivariable-adjusted analysis** AND calculates **Population Attributable Risk (PAR%)**<br>**2 Points**: Reports multivariable-adjusted analysis only<br>**1 Point**: Conducts univariate analysis only
4. Result Consistency	25%	**2 Points**: The direction and significance of the association are **consistent with the conclusions of >50% of the included high-quality studies** (defined as NOS score ≥ 7).<br>**1 Point**: The evidence is **inconsistent or contradictory** to the majority of high-quality findings.

Table S2: Importance Scoring Rubric (0-10 points)

This score assesses the clinical and public health relevance of a given risk factor.

Calculation: Weighted Raw Score = (Guideline Recommendation Score × 0.40) + (PAR% Score × 0.30) + (Intervention Feasibility Score × 0.30)

Final Standard Score(0-10) = 5.0 × (Weighted Raw Score - 1.0)

The multiplier 5.0 linearly scales the score, mapping a theoretical raw score range of [1.0, 3.0] to the standard range of [0, 10].

Dimension	Weight	Scoring Criteria (Original Points)
1. Guideline Recommendation Strength	40%	**3 Points**: Listed as a Class I Recommendation in major national and international cardiovascular prevention guidelines (e.g., Chinese guidelines for cardiovascular disease prevention, ACC/AHA, ESC).<br>**2 Points**: Listed as a **Class IIa Recommendation**.<br>**1 Point**: Not explicitly listed, or listed as **Class IIb/III**.
2. Population Attributable Risk (PAR%)	30%	**3 Points**: PAR% > 20%<br>**2 Points**: PAR% 10% – 20%<br>**1 Point**: PAR% < 10%<br>*Note: If PAR% data were unavailable, the score was assigned based on a pre-defined consensus between reviewers, considering the magnitude of the odds ratio (OR) and the epidemiological significance of the factor.*
3. Intervention Feasibility	30%	**3 Points**: **Easily modifiable** at the population level via established, cost-effective interventions (e.g., smoking cessation programs).<br>**2 Points**: **Modifiable with moderate difficulty**; effective interventions exist but face implementation challenges (e.g., long-term medication adherence for hypertension).<br>**1 Point**: **Lacks effective population-level interventions** or feasibility is currently low (e.g., non-modifiable genetic risk factors).

Example: Practical Scoring Demonstration for a Single Risk Factor

Risk Factor: Current Smoking

Step 1: Evidence Strength Scoring

- **Study Design:** Prospective Cohort Study → **3 Points**
- **Sample Size:** > 1000 cases → **3 Points**
- **Methodological Rigor:** Multivariable analysis with PAR% reported → **3 Points**
- **Result Consistency:** Findings consistent with >50% of high-quality studies → **2 Points**

Weighted Raw Score = (3 × 0.30) + (3 × 0.20) + (3 × 0.25) + (2 × 0.25) = 0.9 + 0.6 + 0.75 + 0.5 = **2.75**

Final Standard Score = 5.714 × (2.75 - 1.0) = 5.714 × 1.75 = **10.0 points**

Step 2: Importance Scoring

- **Guideline Recommendation:** Class I Recommendation → **3 Points**
- **PAR%:** > 20% → **3 Points**
- **Intervention Feasibility:** Easily modifiable (e.g., cessation programs) → **3 Points**

Weighted Raw Score = (3 × 0.40) + (3 × 0.30) + (3 × 0.30) = 1.2 + 0.9 + 0.9 = **3.0**

Final Standard Score = 5.0 × (3.0 - 1.0) = 5.0 × 2.0 = **10.0 points**

Conclusion: The calculated coordinates for **Current Smoking** are **(10.0, 10.0)**. This places it in **Quadrant I (High Evidence Strength – High Importance)** of the matrix, designating it as a **core priority for immediate public health intervention**.
