# Supplementary material for: Current status of premature myocardial infarction research in China: a systematic review and international comparative perspective
Source: Front Cardiovasc Med. 2026 Jul 28;13:1858728. doi: 10.3389/fcvm.2026.1858728 (PMC13457063; doi:10.3389/fcvm.2026.1858728)
Supplement: Supplementary file 2 [file Datasheet2.pdf]

Supplementary Table S3. Raw and Standardized SIM Scores for PMI Risk Factors in China (n=13)

Risk Factor	No. of Studies[1]	Est. PMI Case-Records[2]	Evidence Strength (Raw)	Evidence Strength (Std 0–10)	Importance (Raw)	Importance (Std 0–10)	SIM Quadrant
Smoking	298	28500	2.8	10.0	3.0	10.0	I
Hypertriglyceridemia	215	19200	2.6	9.1	3.0	10.0	I
Family History	145	12500	2.5	8.3	1.7	8.0	I
Metabolic Syndrome	180	16000	2.5	8.6	2.0	5.0	I
Psychological Stress	115	10500	1.8	4.3	2.0	5.0	II
Overwork	110	9800	1.8	4.3	2.0	5.0	II
Sleep Deprivation	95	8500	1.8	4.3	2.0	5.0	II
Gene-Environment Interaction	40	3500	1.5	2.9	1.3	6.0	II
Hypertension	255	23800	2.8	10.0	2.3	4.0	IV
Diabetes Mellitus	220	20100	2.8	10.0	2.3	4.0	IV
Chronic Kidney Disease	85	7200	1.8	6.8	1.7	3.5	IV
Inflammatory Markers	70	6000	1.5	2.9	1.3	1.5	III
Novel Biomarkers	60	5500	1.5	2.9	1.3	1.5	III

[1] No. of Studies: Counted from the 317 included studies reporting the specific risk factor.

[2] Est. Factor-Exposure Records: Represents the sum of PMI cases across studies reporting the specific risk factor. This metric reflects the total volume of factor-specific evidence rather than unique patient counts (i.e., patients with multiple risk factors are counted repeatedly across different factor categories).SIM Quadrant: Assigned based on median cutoffs (Evidence ≥ 6.5; Importance ≥ 5.0).

SIM Quadrant: Assigned based on the median split of Evidence Strength and Importance scores (Median Evidence = 6.5; Median Importance = 5.0) to generate the four-quadrant matrix in Figure 2. Factors scoring above the median in both dimensions were classified as Quadrant I.

Note: The threshold of ≥6.5 (corresponding to 'High-quality' evidence in GRADE) was considered during sensitivity analysis but was not used for the primary quadrant assignment to avoid arbitrary exclusion of contextually important factors.

Supplementary Table S4. Justification for Weighting Choices in the Strength-Importance Matrix (SIM)

SIM Dimension	Weight	Rationale (based on GRADE & Oxford CEBM)
**Evidence Strength**		
Study Design	30%	Prioritizes minimization of bias and causal inference strength.
Sample Size	20%	Larger samples reduce random error.
Methodological Rigor	25%	Reflects adjustment for confounders and outcome validity.
Consistency	25%	Increases confidence when findings are replicated.
**Public Health Importance**		
Guideline Strength	40%	Direct reflection of clinical actionability.
PAR%	35%	Measures potential population-level impact.
Intervention Feasibility	25%	Assesses real-world implementability.

Note: The weights listed above determine the Raw Scores (0–3 for Evidence, 0–3 for Importance). These raw scores are then linearly transformed to a 0–10 scale (as detailed in Appendix S1). For the purpose of quadrant classification in Figure 2, cutoff points of ⩾ 6.5 for Evidence Strength and ⩾ 5.0 for Importance were applied, corresponding to the upper range of moderate-to-high quality evidence and the threshold for clinical relevance, respectively.
