# Supplementary material for: Current status of premature myocardial infarction research in China: a systematic review and international comparative perspective
Source: Front Cardiovasc Med. 2026 Jul 28;13:1858728. doi: 10.3389/fcvm.2026.1858728 (PMC13457063; doi:10.3389/fcvm.2026.1858728)
Supplement: Supplementary file 3 [file Datasheet3.pdf]

Supplementary Table S5. Summary of characteristics and key findings of the 317 included studies.

NO	First author	Journal title	Publication date	Study design	Sample size (n)	Conclusion	Quality assessment (NOS score)
1	Yan Mingzhou	Journal of Norman Bethune University of Medical Science	1999	Cross-sectional Study	33	Among young AMI patients, coronary pathology and risk factors escalated with age. Although smoking and hazardous drinking dominated, traditional risks like hypertension and diabetes were uncommon. Most events were triggered by acute triggers, such as binge smoking, alcohol abuse, emotional stress, or overwork	4
2	Feng Xiaoxia	J Guangdong Med Coll	2000	Retrospective Case-Control Study	53	Young AMI patients are predominantly mental workers with a high smoking rate. They often lack prodromal symptoms and typically present with single-vessel disease.	4
3	Qing Guozhong	China Journal of Mordern Medicine	2001	Retrospective Cohort S	60	Acute myocardial infarction (AMI) in patients under the age of 45 is closely associated with smoking and heavy alcohol consumption, followed by a positive family history. The onset of the disease is often triggered by clear precipitating factors. Normal coronary arteries and single-vessel lesions are common, with generally mild disease severity. Coronary artery spasm and thrombosis may be important underlying causes.	4
4	Sun Baihua	Journal of Heze Medical College	2001	Retrospective Case-Co	204	Smoking (especially heavy smoking), hyperlipidemia, and a family history of myocardial infarction are significant contributing factors to MI in young adults. In contrast, hypertension, diabetes mellitus, alcohol consumption, and physical inactivity show no significant association with MI in this population.	6

5	Jiao Hua	Chin. J. Behav. Med. Sci.	2001	Retrospective Cohort S	240	The incidence of AMI is higher among middle-aged and young males, who frequently have a family history of coronary heart disease (CHD) or hypertension. The infarction predominantly involves the anterior and anteroseptal walls, accompanied by typical clinical symptoms, fewer complications, and a favorable prognosis.	6
6	Wang Lei	Journal of Clinical and Experimental Medicine	2002	Retrospective Cohort S	148	Smoking and hyperlipidemia are high-risk factors for young patients with acute myocardial infarction (AMI). These patients predominantly present with single-vessel disease, and early percutaneous coronary intervention (PCI) yields better clinical benefits.	6
7	Yang Xiaoduan	Practical Clinical Medicine	2002	Retrospective Case-Co	80	The prevalence of coronary heart disease (CHD) risk factors was significantly higher in the young AMI group than in the elderly group. In contrast, triglyceride levels and precipitating factors were significantly higher in the elderly group compared with the young group. Furthermore, complications were more frequent and more severe in the elderly group.	4
8	Zhang ZhaoXia	Ningxia Med J	2002	Retrospective Case-Co	76	Smoking is a significant risk factor for coronary artery lesions in young men.	4
9	Gao Xue	Ningxia Med J	2002	Retrospective Case-Co	42	Smoking and a positive family history are the major risk factors for acute myocardial infarction (AMI) in young adults. The clinical presentation of AMI in this population is predominantly chest pain, accompanied by few complications. Furthermore, thrombolytic therapy yields a high recanalization rate with minimal treatment-related complications, resulting in a favorable prognosis.	4
10	Shao Lei	Henan Journal of Diagnosis and Therapy	2002	Retrospective Case-Co	160	Excess smoking excess drinking fatigue and mental strain are probably the most important risk factors of a cute myocardial infarction.	6

11	Zhang Yuelan	Chinese Journal of Cardiovascular M edicine	2003	Retrospective Cohort S	325	The onset of the disease in the young group is associated with risk factors such as smoking and dyslipidemia, whereas the prime-aged group is associated with risk factors including dyslipidemia, smoking, and hypertension.	6
12	Li Minfeng	Chin. J. Geriatr. Heart Brain Vessel Dis	2003	Cross-sectional Study	81	Young and elderly patients with acute myocardial infarction (AMI) exhibit distinct characteristics in terms of risk factors and psychological manifestations. AMI in young patients is closely associated with smoking and heavy alcohol consumption, among which smoking is the most significant risk factor for this group.	4
13	Jin Qizhi	Zhejiang Practicle Medicine	2003	Retrospective Cohort S	126	Young patients with AMI have a predilection for anterior wall infarction. Normal coronary angiography findings and mild lesions are common in this population. It is hypothesized that coronary artery spasm, triggered by endothelial dysfunction, heavy smoking, and stress factors, is one of the important mechanisms underlying AMI in young adults.	4
14	Li Yuan	Guangdong Medical Journal	2003	Retrospective Case-Co	56	Smoking is the most prominent risk factor for AMI in young adults, particularly heavy smoking. In contrast, hypertension and diabetes mellitus are the two primary risk factors for the disease in the elderly population.	4
15	Yuan Yongsheng	Hebei Medical Journal	2003	Retrospective Case-Co	107	Smoking ranks as the leading risk factor for AMI in the young population, and smoking cessation can effectively reduce the incidence of AMI among young individuals.	6
16	Li Qingshan	ACTA ACADEMIAE MEDICINAE QINGDAO UNIVERSITATIS	2004	Retrospective Case-Co	105	Heavy smoking and a positive family history are major risk factors for acute myocardial infarction (AMI) in young individuals. Coronary artery spasm may play a crucial role in the pathogenesis of AMI in this population.	6

17	Sun Yun	Journal of Henan Medical college for Staff and Workers	2004	Retrospective Cohort S	130	Excessive smoking, overexertion, and mental stress are significant risk factors for the onset of acute myocardial infarction (AMI) in young adults.	6
18	Fu Xiaohua	Clinical FOCUS	2004	Cross-sectional Study	187	Family history of coronary heart disease, smoking, and male sex constituted the primary risk factors for acute myocardial infarction in young and middle-aged adults.	4
19	Wang Jianhua	Chinese Journal of Cardiology	2004	Retrospective Case-Co	497	Young AMI is strongly associated with male gender and smoking, predominantly presenting as single-vessel coronary artery disease. With advancing age, there is a rising trend in the prevalence of multivessel disease, the burden of cardiovascular risk factors, and mortality rates.	6
20	Gao Haiyan	Chin J Cardiovasc Rev	2004	Retrospective Case-Co	103	Young AMI patients are predominantly male, with smoking and alcohol consumption rates reaching 95.5% and 90.9%, respectively. In contrast, hypertension and diabetes are prevalent among elderly AMI patients. Psychologically, young patients primarily exhibit anxiety, while elderly patients are more prone to depression.	4
21	Ma Xiaochuan	J Yan'an Univ (Med Sci)	2004	Retrospective Case-Co	160	The young AMI group predominantly lacks a history of hypertension or diabetes mellitus but exhibits a high smoking rate (92.5%). Disease onset is frequently triggered by precipitating factors such as excessive smoking, overwork, and psychological stress. Coronary angiography primarily reveals single-vessel disease	6
22	Wang Fengqin	Chinese Journal of Integrative Medicine on Cardio-/Cerebrovascular Diseas	2004	Retrospective Case-Co	112	Male patients predominated among young myocardial infarction patients. Smoking, staying up late, and a family history of premature coronary heart disease were independent risk factors for acute myocardial infarction in the young group.	5
23	Xu Guangma	Guangxi Medical Journal	2004	Retrospective analysis	21	Smoking, hypercholesterolemia, hypertension, overweight, and family history of CHD constituted the primary risk factors for AMI in young adults	4

24	Lin Zhaoheng	Medicine and Pharmacy of Yunnan	2005	Retrospective Case-Co	66	Hypertension, diabetes mellitus, and hyperlipidemia are well-established risk factors for coronary heart disease (CHD). The impact of these risk factors on the initiation and progression of coronary atherosclerosis typically requires a prolonged period. In contrast, smoking and a positive family history are the primary risk factors for acute myocardial infarction (AMI) in young patients.	4
25	Wu Hongmei	Journal of Postgraduates of Medicine	2005	Cross-sectional Study	55	The increasing incidence of AMI in young males is associated with smoking and dyslipidemia. With advancing age, multi-vessel and complex coronary artery lesions become more prevalent.	4
26	Zhang Aizhen	Chinese Journal of Cardiology	2005	Retrospective Cohort S	103	Male gender, excessive smoking, and heavy alcohol consumption are the primary risk factors for young patients with AMI, whereas comorbidities such as hypertension and diabetes mellitus constitute the main risk factors for the elderly. Clinically, young patients predominantly exhibit anxiety accompanied by typical chest pain, while elderly patients are characterized by depression, atypical pain symptoms, higher rates of complications, and increased mortality.	6
27	Pang Xia	Journal of Postgraduates of Medicine	2005	Retrospective Case-Co	55	The onset of AMI in young adults is associated with male gender, smoking, and dyslipidemia.	4
28	Liu Jieyun	Journal of Henan University(MedicalScience)	2005	Retrospective Case-Co	140	Heavy smoking, excessive alcohol consumption, a family history of coronary heart disease, and obesity are prominent risk factors for myocardial infarction in young adults. Unhealthy lifestyle habits combined with genetic factors accelerate the early formation of coronary artery lesions.	6

29	Zhang Chengli	People's Mil Surg	2005	Retrospective Case-Cc	101	The predominant risk factors for young AMI patients include smoking (93.1%), overweight (77.2%), and dyslipidemia (71.3%). Single-vessel coronary artery disease is commonly observed (61/90), leading to favorable outcomes following interventional therapy.	5
30	Ma Hong	Inner Mongolia Med J	2005	Retrospective Case-Cc	70	Heavy smoking is the predominant risk factor for AMI in young adults. Coronary artery spasm is frequently observed in this population, often in the absence of traditional risk factors such as hypertension, diabetes mellitus, and hyperlipidemia. Consequently, smoking cessation remains the primary preventive measure.	4
31	Li Jing	Chin J Mult Organ Dis Elderly	2006	Retrospective Cohort S	1056	Elderly patients with myocardial infarction require enhanced monitoring and control of blood pressure and blood glucose, whereas young and middle-aged patients should place greater emphasis on modifying unhealthy lifestyles.	7
32	Song Shifei	Med Theor & Prac	2006	Retrospective Cohort S	100	Male sex, a family history of premature coronary heart disease (CHD), and heavy smoking are the major risk factors for acute myocardial infarction (AMI) in young patients.	4
33	Han Yong	Shaanxi Medical Journal	2006	Retrospective Case-Cc	122	Age and sex are closely correlated with the incidence of acute myocardial infarction (AMI). In young patients, AMI is frequently associated with smoking and elevated triglycerides, and single-vessel coronary artery disease is the predominant pattern. The risk factors and coronary artery lesions in AMI patients vary across different age groups; therefore, not only should comprehensive interventions be implemented for multiple risk factors, but targeted measures should also be applied based on specific patient characteristics.	5

34	Jiang Jianqiang	Med J West China	2006	Retrospective Cohort !	88	Compared with the elderly group, young patients exhibit significantly higher rates of precipitating factors, primary clinical manifestations, family history, and a history of smoking and alcohol consumption. However, the prevalence of hypertension, hyperlipidemia, diabetes mellitus, prior myocardial infarction (MI), cardiogenic shock, and heart failure is significantly lower in this population.	4
35	Wang Qiufen	Journal of Qiqihar Medical College	2006	Retrospective Case-Cc	120	Compared with the elderly group, young patients exhibit significantly higher rates of precipitating factors, primary clinical manifestations, family history, and a history of smoking and alcohol consumption. However, the prevalence of hypertension, hyperlipidemia, diabetes mellitus, prior myocardial infarction (MI), cardiogenic shock, and heart failure is significantly lower in this population.	6
36	Li Weiju	Chinese Remedies & Clinics	2006	Retrospective Case-Cc	112	Acute myocardial infarction (AMI) in young adults predominantly affects males. The primary precipitating factors include excessive physical exertion, emotional agitation, and heavy alcohol consumption, while smoking and a family history serve as the main risk factors. Clinically, these patients present with severe symptoms but have fewer complications, resulting in a low mortality rate and a favorable prognosis.	5
37	Gao Meng	Nurs J Chin PLA	2006	Retrospective Cohort !	212	The precipitating factors and risk factor profiles differ distinctly between elderly and young patients with AMI. The elderly group is characterized by multiple comorbidities that interact with each other, with hypertension being the most prominent. In contrast, the risk factors in young adults are primarily driven by severe unhealthy lifestyle habits, among which smoking is the most prevalent.	6

38	Guo Yujun	Clinical Focus	2006	Retrospective Case-Co	341	Male gender, excessive smoking, and heavy alcohol consumption are the primary risk factors for young patients with AMI, whereas comorbidities such as hypertension and diabetes mellitus constitute the main risk factors for the elderly. Clinically, young patients predominantly exhibit anxiety accompanied by typical chest pain, while elderly patients are characterized by depression, atypical pain symptoms, higher rates of complications, and increased mortality.	7
39	Xiao Ling	Chin J Arterioscler	2006	Retrospective Case-Co	76	Acute myocardial infarction (AMI) in young adults shows a male predominance, with distinct risk and precipitating factors. Symptoms are typically classic, coronary lesions are predominantly single-vessel, and complications are rare; consequently, early interventional therapy yields a favorable prognosis. Furthermore, implementing standardized secondary prevention for CAD in this population is crucial to preventing the premature onset of AMI.	7
40	Xu Rong	Chinese Journal of Cardiovascular Review	2006	Retrospective Cohort S	378	Young patients may also present with gastrointestinal symptoms as the initial manifestation of AMI. The primary risk factor in this group is hyperlipidemia, whereas other age groups frequently exhibit a coexistence of hypertension and hyperlipidemia.	7

41	Guo Yujun	Clinical Focus	2006	Retrospective Case-Co	341	The incidence of AMI in young adults is on an upward trend. Compared to elderly patients, young AMI exhibits a significant gender disparity, with males outnumbering females. Smoking and family history are critical risk factors. Clinical symptoms are more typical than those observed in elderly patients. Coronary angiography predominantly reveals single-vessel disease, frequently involving the left anterior descending (LAD) artery; double- and multi-vessel diseases are less common than in the elderly group. Furthermore, young patients experience fewer and milder complications, resulting in lower mortality rates and a favorable prognosis.	7
42	Liu Dongxia	Clinical Medicine of China	2006	Retrospective Case-Co	317	Smoking (OR=2.608), alcohol consumption (OR=2.844), overweight/obesity (OR=3.682), family history of premature coronary heart disease (OR=4.265), and lack of physical exercise (OR=2.773) are independent risk factors for AMI in middle-aged and young patients, whereas hypertension is associated with AMI in the elderly.	6
43	Zhu Haixia	Inner Mongolia Med J	2006	Retrospective Case-Co	102	Excessive alcohol consumption, overexertion, and mental tension may serve as potential precipitating factors for acute myocardial infarction in young adults.	6
44	Han Lanquan	Clinical Medicine	2007	Retrospective Case-Co	83	The main precipitating factors for acute myocardial infarction in young patients are smoking, hypercholesterolemia, and hypertension, while the underlying etiology is primarily coronary atherosclerosis.	4
45	Gao Junfeng	Central Plains Medical Journal	2007	Retrospective Case-Co	114	Atherosclerosis is the basic pathogenic factor of the youthful AMI. Smoking, hpertention, hypercholesterolemia are mainly risk factor.	5

46	Zhai Yunzhong	Huaxia Journal of Medicine	2007	Retrospective Case-Cc	124	Young patients with acute myocardial infarction (AMI) present with typical clinical manifestations. Smoking is their primary risk factor, and they have fewer complications, lower mortality rates, and a more favorable prognosis.	5
47	Liu GuangPing	Xinjiang Medical Journal	2007	Retrospective Case-Cc	74	The primary risk factors for acute myocardial infarction (AMI) in young patients, in descending order, are smoking, hyperlipidemia, a family history of cardiovascular disease, and hypertension. Smoking may be the most significant risk factor for AMI in this population.	4
48	Sun Shengli	Chinese and Foreign Medical Research	2007	Retrospective Cohort S	157	Smoking, hyperlipidemia, and a family history of coronary heart disease (CHD) are the primary risk factors for AMI in young adults. Lipid-lowering therapy, smoking cessation, and adopting healthy work and lifestyle habits are fundamental measures to reduce the incidence of AMI in this population. Furthermore, reperfusion therapy yields favorable outcomes in young patients with AMI.	6
49	Wang Zhe	Clinical Medicine of China	2007	Cross-sectional Study	85	Acute myocardial infarction (AMI) predominantly affects middle-aged and young adults, especially males. These patients frequently present with risk factors such as smoking and alcohol consumption, often maintain unhealthy lifestyles, and typically experience an acute onset triggered by identifiable precipitating factors.	4
50	Li Yuansheng	Fujian Med J	2007	Retrospective Case-Cc	72	Young AMI patients exhibit a high prevalence, large volume, and frequent pattern of alcohol consumption, suggesting that long-term excessive drinking is a significant risk factor for AMI in this population. Additionally, a positive family history of coronary artery disease (CAD) represents another crucial risk factor.	5

51	Cai Xueli	Zhejiang Medical Journal	2007	Retrospective Case-Co	154	Heavy smoking, excessive psychological stress and fatigue, heavy alcohol consumption, and a family history of cardiovascular disease are significant risk factors for AMI in young adults. Therefore, lifestyle modifications such as smoking and alcohol cessation, emotional regulation, adequate relaxation, and avoiding overexertion are effective measures to prevent AMI in this population.	5
52	Huang Conggang	Chin J Epidemiol	2007	Retrospective Case-Co	242	Young AMI is associated with a cluster of risk factors, including smoking, dyslipidemia, family history, and precipitating factors such as overwork and heavy smoking. Coronary angiography predominantly reveals single-vessel disease.	6
53	Li Yuezhou	J Pract Diagn Ther	2007	Retrospective Case-Co	81	Young AMI is closely associated with smoking and heavy alcohol consumption, with anxiety being the predominant psychological manifestation. In contrast, hypertension and diabetes mellitus are the primary risk factors in the elderly group, where depression is the predominant psychological feature	5
54	Zhao Hua	Pract J Cardiac Cereb Pneumal Vasc Dis	2007	Retrospective Case-Co	45	Among rural young AMI patients, males predominate, with a smoking rate of 91.1% and precipitating factors such as heavy drinking or overwork reaching 95.6%. These patients typically present with a paucity of prodromal symptoms and predominantly single-vessel disease. Notably, coronary angiography (CAG) results are normal in 38.1% of cases, and the overall prognosis is favorable.	5
55	Xia kun	Shandong Medical Journal	2007	Retrospective analysis	32	Unhealthy lifestyle habits, particularly smoking, appear to be prominent contributing factors for myocardial infarction in young adults. Although hypertension and diabetes are established risk factors for coronary heart disease, they accounted for a relatively small proportion in our cohort and did not predominate among the risk factors for premature AMI.	4

56	Yuan Yongsheng	Zhejiang Practical Medicine	2007	Retrospective Case-Co	107	The major risk factors for young acute myocardial infarction ranked in descending order were smoking, hyperlipidemia, and positive family history. Mitigating these adverse lifestyle habits would be beneficial in reducing the incidence of AMI	5
57	Wang Yunfei	Journal of Clinical Emergency	2008	Retrospective Case-Co	195	The incidence of acute myocardial infarction (AMI) in young people under 40 is significantly higher in males than in females. Smoking, heavy alcohol consumption, hyperlipidemia, and family history are the primary risk factors. The onset is typically characterized by classic symptoms, with fewer complications, a lower mortality rate, and an overall favorable prognosis.	5
58	Zhang Ping	Prevention and Treatment of Cardio-Cerebra-l Vascular Disease	2008	Retrospective Cohort S	97	The primary etiology of acute myocardial infarction (AMI) is coronary atherosclerosis. In young patients, coronary artery lesions are generally less severe and predominantly single-vessel, with smoking and family history being the main risk factors. Conversely, elderly patients mostly present with multi-vessel disease and multiple comorbidities, resulting in a poorer prognosis, with hypertension as their primary risk factor. There was no significant difference in the degree of elevated blood lipids between the two groups.	6
59	Ren Huimin	Qingdao Medical Journal	2008	Retrospective Cohort S	270	Acute myocardial infarction (AMI) predominantly affects males in the young and middle-aged population. Distinctive features of this group, compared to the elderly, include smoking, alcohol consumption, family history, and elevated triglycerides (TG). The disease typically presents with a sudden onset and classic chest pain. Patients generally have a favorable prognosis, accompanied by lower rates of complications and mortality. Furthermore, coronary angiography reveals that single-vessel lesions are predominant, while three-vessel disease is relatively rare.	6

60	Li Jiwu	Chin J Mod Drug Appl	2008	Retrospective Cohort S	80	Smoking is the most significant risk factor for young patients with acute myocardial infarction (AMI), and their coronary artery disease is predominantly characterized by single-vessel lesions. Smoking cessation, alcohol restriction, avoidance of excessive fatigue, and maintaining a positive mindset are crucial measures to reduce the incidence of AMI in young individuals.	5
61	Zhang Weiping	J South Med Univ	2008	Retrospective Case-Co	491	Smoking, metabolic disorders, and a positive family history are the major risk factors for acute myocardial infarction (AMI) in patients under 40 years of age. The severity of coronary artery lesions is significantly less severe in young AMI patients compared to the elderly group, and changes in HDL-C play a vital role in determining the degree of coronary stenosis in young individuals.	5
62	Sun ChangXi	Shandong Medical Journal	2008	Retrospective Cohort S	100	The young group has higher proportions of smoking, high mental stress or extreme fatigue, and a family history of cardiovascular disease compared to the elderly group. Therefore, measures such as smoking cessation, emotional regulation, and avoiding excessive fatigue are effective strategies for preventing AMI in young patients.	6
63	Wang BaoFu	Journal of Chinese Physician	2008	Retrospective Cohort S	100	Young patients with acute myocardial infarction (AMI) often have clear precipitating factors or risk factors such as smoking and a family history of coronary heart disease. Controlling these triggers and risk factors is crucial for young AMI patients.	5

64	Zhang Hong	Chinese JoumaI of MedicinaI Guide	2008	Retrospective Case-Co	56	Smoking, excessive alcohol consumption, hyperlipidemia, hypertension, a family history of coronary heart disease, and diabetes are risk factors associated with AMI. It is recommended to quit smoking, eliminate unhealthy lifestyle habits, maintain a positive mindset, adhere to a balanced diet, and avoid excessive fatigue. Early detection and control of risk factors such as hypertension, hyperlipidemia, and diabetes, along with preventive monitoring for high-risk individuals with genetic predispositions, are of great significance in preventing AMI in young adults.	4
65	Liu Yinghua	JOURNAL OF PRACTICALEI CTROCARDIOLOG Y JS	2008	Retrospective Cohort S	236	Common risk factors include smoking, excessive alcohol consumption, hypertension, hyperlipidemia, obesity, diabetes mellitus, and a family history of coronary heart disease. Among these, smoking and excessive alcohol consumption are the primary precipitating factors for AMI in middle-aged and young adults.	7
66	Jin Jing	Proceeding of Clinical M edicine	2008	Retrospective Cohort S	221	The young group of AMI are all men, female age of onset later, AMI youth group closely related to the incidence of smoking. The rate of coronary artery with more complex lesions increased with the growth of age.	6
67	Zhang Jianjiang	Prevention and Treatment of Cardio-Cerebra-l Vascular Disease	2008	Retrospective Cohort S	156	The prevalence of two risk factors, a family history of coronary heart disease (CHD) and smoking, was significantly higher in the middle-aged and young group compared to the elderly group. In contrast, the incidences of hypertension and diabetes mellitus were significantly lower than those in the elderly group. No significant differences were observed between the two groups regarding novel risk factors such as C-reactive protein (CRP), fibrinogen (Fib), and lipoprotein(a) [Lp(a)].	6

68	Wang Cui	Journal of Military Surgeon in Southwest China	2008	Retrospective Case-Co	84	Smoking, a family history of the disease, obesity, hyperlipidemia, hypertension, and diabetes mellitus are underlying risk factors for acute myocardial infarction (AMI) in middle-aged and young adults. Furthermore, 85% of these patients had identifiable precipitating factors, such as overwork, emotional agitation, and excessive alcohol consumption.	4
69	Fang Jianmei	Nurs J Chin PLA	2008	Cross-sectional Study	217	Early nursing intervention could effectively improve patients’knowledge about their disease and abilities of self health care． which plays a vital role in preventing occurrence and developemtn of AMI in youth．	4
70	Liu Yi	Captital Medicine	2008	Retrospective Case-Co	89	Acute myocardial infarction (AMI) in young patients is predominantly associated with smoking and hypertriglyceridemia. The onset often has clear precipitating factors, such as heavy alcohol consumption and excessive physical exertion. Coronary artery lesions are mainly characterized by single-vessel disease.	4
71	Gong Haiying	Chinese Journal of Cardiovascular Medicine	2008	Retrospective Case-Co	199	Smoking and drinking are two main causes of AMI in young patients· T he removal of risk factors and timely reperfusion therapy are of importance to reduce morbidity and mortality and to improve prognosis·	6
72	Fang Jianmei	Nurs J Chin PLA	2008	Retrospective Case-Co	154	Male gender, excessive smoking, heavy alcohol consumption, and high stress levels are the primary risk factors for AMI in young adults. Early nursing interventions should be implemented to enhance patients' disease awareness and self-care capabilities, thereby preventing the onset and progression of AMI.	6

73	Xie Zhijuan	Med J West China	2008	Retrospective Case-Cc	73	Male gender, a family history of premature coronary heart disease, heavy smoking (OR=14.006), heavy alcohol consumption (OR=5.556), and elevated homocysteine (Hcy) levels (OR=1.554) are independent risk factors for AMI in middle-aged and young patients. Folic acid supplementation has been shown to effectively reduce Hcy levels.	6
74	Tian Ling	Chin J Mult Organ Dis Elderly	2009	Retrospective Cohort !	218	Heightened attention should be paid to female AMI patients, with active control of traditional risk factors to prevent and intervene in atherosclerosis. Furthermore, close attention should be directed toward the roles of genetic factors, coronary artery spasm, endothelial dysfunction, and hypercoagulable state in the pathogenesis of myocardial infarction.	5
75	Wang Haiyu	Chinese Journal of Cardiovascular Research，	2009	Retrospective Case-Cc	190	Smoking, genetic predisposition, and low high-density lipoprotein cholesterol (HDL-C) are the most significant risk factors for young patients with ST-segment elevation myocardial infarction (STEMI). Coronary artery lesions in young acute STEMI patients are predominantly of low-to-intermediate risk, mostly involving the left anterior descending (LAD) artery. Aggressive control of these risk factors, combined with timely percutaneous coronary intervention (PCI) for reperfusion therapy, is of paramount importance in reducing disease incidence and improving prognosis.	5
76	Cai Xueli	Journal of Wenzhou Medical College	2009	Retrospective Case-Cc	217	Male， smoking， clear incentive and alcoholism were the main risk factors in the Youth AMI patients. Youth AMI patients were the clinical manifestations of sudden angina pectoris， less complications， low mortality and favorable prognosis. Good habits， life style and timely reperfusion therapy are of great significance to prevent disease and to improve prognosis.	6

77	Zhang HongYu	North China Coal Medical University	2009	Retrospective Cohort S	80	Young patients with acute myocardial infarction (AMI) present with typical clinical manifestations and multiple risk factors for coronary heart disease. They typically have a mild degree of coronary artery stenosis, fewer complications, and a favorable prognosis.	6
78	Zhang JinQian	China Healthcare Frontiers	2009	Retrospective Case-Cc	148	Smoking and a family history are the primary risk factors in young patients, with acute myocardial infarction (AMI) being more prevalent in males. The incidence of heart failure is relatively low in this population; therefore, timely reperfusion therapy is crucial for reducing mortality and improving prognosis.	6
79	Shen Chong	Chinese Journal of Medicinal Guide	2009	Retrospective Cohort S	148	A history of smoking, a family history of the disease, and a high body mass index (BMI) are risk factors for acute myocardial infarction (AMI) in middle-aged and young adults."	6
80	Xiao Shigui	Journal of Chinese Physician	2009	Retrospective Case-Cc	125	The primary characteristics of AMI in young adults include male predominance, smoking, hyperlipidemia, and obesity. Coronary artery lesions are typically uncomplicated, with distinct precipitating factors identified.	6
81	Hou Kaihuan	Guide of China Medicine	2009	Retrospective analysis	60	Young AMI patients show a male predominance, with excessive alcohol and tobacco consumption often serving as precipitating factors. A family history of coronary atherosclerotic heart disease (CAHD) and hyperlipidemia may be more prominent risk factors for the premature onset of AMI.	4
82	Li Jianwen	Int Med & Health Guidance News	2009	Retrospective Case-Cc	135	Young and middle-aged AMI patients are characterized by male gender, smoking, alcohol consumption, a positive family history, and elevated triglycerides (TG). In contrast, the elderly population is predominantly driven by hypertension and diabetes mellitus.	6

83	Jiana Ti	Journal of Clinical and Experimental Medicine	2010	Retrospective Case-Co	80	In young patients with AMI, heavy smoking and a positive family history are significant risk factors. Single-vessel disease is common, and the prognosis is generally favorable.	4
84	Li Xiaorong	ACTA UNIVERSITATISMEDICINALISNANJING（NaturalScience）	2010	Retrospective Cohort S	218	Smoking, alcohol consumption, family history, and elevated triglycerides (TG) are the primary risk factors for acute myocardial infarction (AMI) in patients under 40 years of age. The severity of coronary artery lesions in young patients is less pronounced than that in the elderly group. Therefore, it is essential to strengthen health education and promote lifestyle modifications — such as blood pressure control and active smoking cessation—to reduce the incidence of AMI among the young population.	6
85	Ma Hong	Chin J Prim Med Pharm	2010	Cross-sectional Study	302	Smoking is the primary risk factor for acute myocardial infarction (AMI) in young individuals, whereas hypertension and diabetes mellitus are the major risk factors for AMI in middle-aged and elderly patients.	5
86	Li Zhen	Chinese Journal of Clinicians (Electronic Edition)	2010	Retrospective analysis	49	Traditional risk factors for coronary heart disease (CHD) remain significant in young patients with acute myocardial infarction (AMI), with smoking and overweight/obesity being particularly prominent, and a clustering trend of risk factors is evident. Adverse lifestyle habits, such as smoking, overweight/obesity, and high psychological stress, are becoming increasingly significant. Modifying unhealthy lifestyles, increasing physical activity, and maintaining a positive mindset and emotional stability are crucial aspects of controlling CHD risk factors in young individuals.	4

87	Huang Jiang	Chinese Journal of Community Physicians。	2010	Retrospective Case-Cc	102	In young patients, the predominant risk factor is smoking, and there are often clear precipitating factors. Single-vessel disease is common, and their clinical outcomes are more favorable compared to non-young patients.	6
88	Wei YongQiang	Contemporary Medicine，	2010	Retrospective Cohort S	110	Smoking, alcohol consumption, chronic sleep deprivation, and fatigue are major risk factors for acute myocardial infarction (AMI) in young patients. Adopting healthier lifestyle habits may significantly reduce the incidence of AMI in this population. Young patients typically present with simple coronary artery lesions; therefore, percutaneous coronary intervention (PCI) yields favorable outcomes, characterized by fewer complications and a good prognosis.	6
89	Han YiHui	Medical Innovation of China	2010	Retrospective Case-Cc	105	Young individuals with a certain genetic predisposition are more susceptible to coronary heart disease (CHD) under the influence of external environmental factors. Therefore, young people with a positive family history should be considered a target population for CHD prevention.	5
90	Zhang XiaoBo	Proceeding of Clinical M edicine	2010	Retrospective Case-Cc	116	The primary risk factors for acute myocardial infarction (AMI) in young women include hyperlipidemia, hypertension, diabetes mellitus, a positive family history of cardiovascular disease, and concomitant diseases. In contrast, the main risk factors for AMI in young men are smoking, hyperlipidemia, hypertension, a positive family history of cardiovascular disease, and diabetes mellitus.	6
91	Fang ZhiHua	China Practical Medicine	2010	Retrospective Case-Cc	100	Smoking, genetic predisposition, and low-density lipoprotein cholesterol (LDL-C) are the most critical risk factors for ST-segment elevation myocardial infarction (STEMI) in young men. Their coronary artery lesions are predominantly single-vessel disease.	7

92	Ci WeiPing	Journal of Cardiovascular & Pulmonary Diseases	2010	Retrospective Cohort S	380	Males constitute the vast majority of young patients with acute myocardial infarction (AMI). Among young male AMI patients, overweight and smoking are the most prominent risk factors.	5
93	Li Rong	Inner Mongolia Journal of Traditional Chinese Medicine	2010	Retrospective Case-Co	117	Excessive smoking, hypertriglyceridemia, overexertion, and mental stress may be significant risk factors for acute myocardial infarction (AMI) in young adults. Coronary artery lesions in this population are predominantly characterized by single-vessel stenosis, often accompanied by severe stenosis and frequent coronary artery spasms.	6
94	Li Jing	Chinese Journal of Cardiovascular Medicine	2010	Retrospective Cohort S	614	Smoking and hyperlipidemia are the most common risk factors for myocardial infarction (MI) in young adults. These patients generally present with milder coronary artery lesions and have a favorable short-term prognosis.	7
95	Li Yingxin	Modern Preventive Medicine	2010	Retrospective Cohort S	98	Hyperlipidemia, hypertension, and diabetes mellitus are common risk factors for both young and elderly patients with AMI; however, the young patient group presents with a greater number of risk factors than the elderly group. Coronary angiography findings reveal that single-vessel lesions are more prevalent in young patients compared to their elderly counterparts, whereas double-vessel lesions and triple-vessel or multi-vessel lesions are less frequent.	6
96	Zhang Xiangqun	Chinese Clinical Practical Medicine	2010	Retrospective Case-Co	99	Patients with premature coronary heart disease and NSTEMI exhibit a higher prevalence of severe lesions. Therefore, risk factors must be controlled early, and coronary revascularization should be performed in a timely manner.	5

97	Tu Ailan	Clinical Medical Engineering	2010	Cross-sectional Study	207	Middle-aged and young adults face immense occupational stress, and the accompanying changes in lifestyle and dietary patterns have contributed to an increased incidence of AMI in this demographic. The occurrence of AMI among middle-aged and young adults is the combined effect of multiple risk factors.	5
98	Zhang Caixia	Journal of Weifang Medical University	2010	Retrospective analysis	120	Male gender, smoking, and a family history of premature coronary heart disease are prominent risk factors for AMI in young adults, whereas carotid intima-media thickness (IMT) is an independent risk factor associated with the severity of coronary artery lesions	6
99	Zhao Xin	Chinese Journal of Arteriosclerosis	2010	Retrospective Case-Cc	183	Young female AMI patients exhibit a disproportionately higher prevalence of hypertension, diabetes mellitus, and family history. They predominantly present with multivessel coronary artery disease.	6
100	Zhou Ying	Hebei Medical Journal	2010	Retrospective Case-Cc	243	The prevalence of smoking and obesity is higher in young and middle-aged patients compared to the elderly, whereas the elderly group exhibits a higher incidence of hypertension, diabetes mellitus (DM), multivessel disease, and complications. In contrast, young patients predominantly present with typical chest pain.	6
101	Han Xianqi	Chin J Med Guide	2010	Retrospective Case-Cc	152	Young male AMI patients exhibit a significantly higher prevalence of heavy smoking, alcohol consumption, and premature coronary heart disease family history compared to the elderly group; conversely, hypertension and diabetes mellitus are more prevalent in elderly patients.	6

102	Liu Xifang	Journal of Medical Forum	2010	Retrospective Case-Co	156	The young group had significantly higher family history of coronary heart disease and smoking rates than the elderly group; Hypertension and diabetes were lower than in the elderly group; Young patients had clear triggers, typical chest pain, fewer complications, and a good prognosis.	6
103	Ping Xiaochuan	Chinese Journal of Health Laboratory Technology	2010	Retrospective Case-Co	125	The culprit vessels in both the young group and young male AMI patients were mainly the left anterior descending artery; The smoking rate among females was low, and passive smoking requires attention; This is consistent with the lesion characteristics of young AMI.	6
104	Chang Wenxiong	Shanxi Medical Journal	2010	Retrospective Case-Co	382	In the Yan'an region, premature acute myocardial infarction in young adults was associated with multiple factors, including high smoking rates, high fat intake, elevated postprandial blood glucose, increased serum cholesterol, parental history of cardiovascular disease, low income, and low educational level	6
105	Hu Changxing	Chin J Cardiovasc Rehabil Med	2011	Retrospective Cohort S	126	Heavy smoking and dyslipidemia are the primary pathogenic factors for acute myocardial infarction (AMI) in young patients under 40 years of age. Concurrent diabetes mellitus and hypertension are relatively uncommon, while single-vessel disease is frequently observed. The in-hospital mortality rate is low, resulting in a favorable short-term prognosis.	6
106	Zu Xiaolin	Journal of Cardiovascular & Pulmonary Diseases	2011	Retrospective Case-Co	601	Middle-aged and elderly patients predominantly present with multi-vessel disease, which is often an acute exacerbation of long-standing chronic lesions. In contrast, coronary artery involvement in young AMI patients primarily affects the left anterior descending (LAD) artery, with relatively isolated lesions. Furthermore, passive smoking may play a significant role in the pathogenesis of acute myocardial infarction in young women.	6

107	He JinXia	J Med Theor＆Prac	2011	Retrospective Case-Co	200	Heavy smoking, heavy alcohol consumption, a positive family history of premature coronary heart disease (CHD), and hypertriglyceridemia are clinical risk factors for male patients with premature acute myocardial infarction (AMI).	6
108	Liu Bende	J Clin Cardiol (China)	2011	Cross-sectional Study	40	Smoking, onset in the early morning, lack of physical activity, and sleep deprivation are the primary risk factors for acute myocardial infarction (AMI) in young individuals; whereas underlying conditions such as dyslipidemia and obesity are the risk factors for AMI in patients over 35 years of age.	4
109	Wang Pingxu	Xinjiang Medical Journal	2011	Retrospective Case-Co	116	Young patients with acute myocardial infarction (AMI) mostly have identifiable precipitating factors, predominantly excessive physical exertion or emotional stress. This is closely related to their strong career ambition, intense social competition, and long-term psychological tension and irritability.	5
110	Chen Li	Chinese General Practice	2011	Retrospective Cohort S	389	Smoking and obesity are the most significant risk factors for young patients with acute ST-segment elevation myocardial infarction (STEMI). Coronary artery lesions in young patients are predominantly single-vessel disease, and they experience fewer major adverse cardiovascular events (MACE) during hospitalization compared to elderly patients.	6
111	Zhao Ying	Jilin Medical Journal	2011	Cross-sectional Study	127	Smoking, alcohol consumption, chronic sleep deprivation, and fatigue are major risk factors for acute myocardial infarction (AMI) in young patients. Through nursing interventions, correcting lifestyle habits can significantly reduce the incidence of AMI in this population.	5

112	Wang LiFang	Chinese and Foreign Women Health	2011	Retrospective Case-Cc	157	For the young population, vigorous advocacy for smoking cessation and tobacco control is essential. Strengthening health education and promoting healthy behaviors should be prioritized to reduce precipitating factors such as excessive fatigue and heavy alcohol consumption. For young patients with myocardial infarction, early identification of malignant arrhythmias is crucial to lower both the incidence and mortality rates of acute myocardial infarction in this demographic.	5
113	Zhang Bin	CHINA MEDICINE AND PHARMACY	2011	Retrospective Case-Cc	781	The independent susceptibility factors for myocardial infarction in young patients include age at onset, smoking, and serum leukocyte and blood glucose levels.	6
114	Jing Xin	Med J West China	2011	Retrospective Case-Cc	212	Smoking, male sex, a positive family history, and dyslipidemia are significant risk factors for acute myocardial infarction (AMI) in young adults. The onset is characterized by typical symptoms and few complications. Furthermore, coronary angiography (CAG) reveals normal coronary arteries in a certain proportion of patients.	7
115	Fan Jinying	Chin J Naut Med & Hyperbar Med	2011	Retrospective Case-Cc	46	Body weight ≥80 kg, BMI ≥25, triglycerides (TG) ≥ 1.7 mmol/L, LDL-C ≥3.12 mmol/L, and HDL-C <1.0 mmol/L are the major risk factors for predicting acute myocardial infarction (AMI) in young adults.	4
116	Yang Lei	Chin Med J Metall Indus	2011	Retrospective Case-Cc	92	Young male patients with acute myocardial infarction (AMI) often lack high-risk factors such as hypertension and diabetes mellitus. They typically present with milder coronary artery lesions, predominantly single-vessel stenosis, frequent coronary artery spasms, and sparse collateral circulation. These findings suggest that coronary thrombosis and/or coronary spasm play a pivotal role in the pathogenesis of AMI in this population.	6

117	Zhang Chunming	Medical Innovation of China	2011	Retrospective Cohort !	297	A family history of premature coronary heart disease (CHD), heavy smoking, and alcohol consumption are the primary risk factors for AMI in young patients, whereas comorbidities such as hypertension and diabetes mellitus represent the main risk factors in elderly patients.	5
118	Zhang Caixia	China Modern Medicine	2011	Retrospective Case-Co	120	Male sex, smoking, a family history of premature coronary heart disease (CHD), and C-reactive protein (CRP) levels are prominent risk factors for myocardial infarction in young adults. Meanwhile, the carotid intima-media thickness (CIMT) is an independent risk factor correlated with the severity of coronary artery lesions.	6
119	Xiang Fei	Chin. J. Med. Rec.	2011	Retrospective Cohort !	194	The morbidity of young and middle-aged are mainly male most of them have obvious inducement and typical symptom. Coronary artery lesion used to involve single vascular. Its complication and death ratio is low.	6
120	Cai Jifen	Journal of Kunming Medical University	2011	Retrospective Case-Co	64	The increasing incidence of acute myocardial infarction (AMI) in young adults is largely attributed to unhealthy lifestyle factors, such as smoking, heavy alcohol consumption, physical inactivity, dyslipidemia, and excessive stress.	4
121	Tian Linfang	CHINESE JOURNAL OF INTEGRATIVE MEDICINE ON CARDIO / CEREBROVASCULAR DISEASE	2011	Retrospective Case-Co	89	Risk factors such as smoking, heavy alcohol consumption, hyperlipidemia, and a family history of coronary artery disease are driving factors behind the trend of younger AMI onset. Cultivating healthy lifestyle habits among young adults and implementing timely health education interventions are of great significance for the prevention and control of AMI in this population.	6

122	Qi Hong	Medicine and Pharmacy of Yunnan	2011	Retrospective Cohort S	140	AMI in young adults predominantly affects males, with a male-to-female ratio of approximately 10:1. Prior to onset, young patients frequently present with precipitating factors such as heavy smoking, excessive alcohol consumption, physical overexertion, binge eating, and mental stress. In contrast, the primary precipitating factors for AMI in the elderly include physical overexertion, straining during defecation, large meals, and exposure to cold temperatures.	4
123	Bo Xiaohong	Chinese Journal of General Practice	2011	Retrospective analysis	20	Smoking, excessive alcohol consumption, excessive fatigue, obesity, emotional excitement, family history, and hyperlipidemia are the primary risk factors for acute myocardial infarction (AMI) in young adults.	5
124	Luo Bensheng	Journal of Bengbu Medical College	2011	Retrospective Case-Co	98	he majority of young AMI patients are male and often lack the classic "three highs" risk factors (hypertension, hyperglycemia, and hyperlipidemia), with smoking frequently being the sole risk factor. In non-smokers without these metabolic abnormalities, a family history of premature cardiovascular disease is commonly observed. Compared to middle-aged and elderly AMI patients, young adults exhibit a higher prevalence of single-vessel coronary artery disease.	6
125	Sun Chaoyang	Chin J Misdiagn	2011	Retrospective Case-Co	51	Smoking was the most prominent risk factor for young AMI patients, accounting for 90.2% of cases and ranking first among all risk factors. Coronary artery lesions in young AMI patients were predominantly single-vessel and localized.	6
126	Ye Zhuolian	Jilin Medical Journal	2011	Retrospective Case-Co	182	Smoking and a positive family history are the predominant risk factors. Typical chest pain is common, whereas prodromal angina is rare. Single-vessel coronary artery disease is the predominant pattern.	6

127	Zhang Xianghong	Shandong Medical Journal	2011	Retrospective Case-Cc	188	Young AMI patients are predominantly male, with high prevalence rates of smoking, alcohol consumption, and a positive family history. Most present without prodromal symptoms but exhibit typical chest pain. Coronary artery lesions are predominantly single-vessel disease involving the left anterior descending (LAD) artery, and emergency percutaneous coronary intervention (PCI) yields favorable outcomes.	6
128	Huang Chunyan	Prac J Cardiac Cereb Pneumal Vasc Dis	2011	Retrospective Case-Cc	43	Risk factors for AMI in middle-aged and young patients are predominated by smoking (76.6%), while hypertension, diabetes mellitus, and hyperlipidemia are less common. In contrast, hypertension and diabetes mellitus are the primary risk factors in the elderly group; however, there are no significant age-related differences in the location of myocardial infarction.	6
129	Jiang Jiandang	Chinese Community Doctors	2011	Retrospective Case-Cc	104	Smoking, obesity, hyperlipidemia, hypertension, alcohol consumption, family history, and diabetes mellitus were identified as significant risk factors for young patients with acute myocardial infarction (AMI)	5
130	Luo Jingguang	Chin J Postgrad Med	2012	Retrospective Cohort S	613	Smoking and hyperlipidemia, particularly smoking, are significant risk factors for acute myocardial infarction (AMI) in middle-aged and young patients, playing a crucial role in the trend of earlier AMI onset. Smoking cessation and lipid control hold promise for effectively reducing the incidence of AMI in this population.	6
131	Lu Shiwen	HEBEI MEDICINE	2012	Retrospective Cohort S	114	Hypertension, diabetes mellitus, smoking, alcohol consumption, dyslipidemia, and a family history of premature myocardial infarction (MI) are risk factors for premature MI in women, which significantly affect patient prognosis.	6

132	Yao Kang	Shanghai Medical Journal	2012	Retrospective Cohort S	892	Young patients with acute myocardial infarction (AMI) differ significantly from elderly patients in terms of clinical risk factors, coronary artery lesion characteristics, and prognosis. They are predominantly male, have a higher prevalence of smoking and a family history of coronary heart disease (CHD), and often present with stress-induced precipitating factors such as heavy short-term smoking or intense physical activity. Furthermore, they mostly exhibit single-vessel lesions — particularly thrombotic lesions of the left anterior descending (LAD) artery—which is associated with high success rates of percutaneous coronary intervention (PCI) and favorable long-term prognosis.	6
133	Chen Chunying	Chinese Journal of Community Physicians。	2012	Retrospective Case-Co	60	Smoking， excessive drinking and exercise at onset，less ， less sleep ， is a major risk factor for AMI youth；And high blood pressure，obesity and dyslip idemia based disease is ＞ 35 AMI risk factors.	5
134	Fan RuiJuan	Clinical Medicine of China	2012	Retrospective Cohort S	411	Smoking, alcohol consumption, obesity, family history, and dyslipidemia are the major risk factors for acute myocardial infarction (AMI) in young patients. The onset is often precipitated by overwork or extreme emotional agitation. This group typically presents with fewer prodromal symptoms and has a favorable long-term prognosis.	7

135	Dong ZhenYu	Contemporary Medicine,	2012	Retrospective Case-Co	236	The primary risk factors for acute myocardial infarction (AMI) in young patients, in descending order, are smoking, hyperlipidemia, and a positive family history. The clinical characteristics of young AMI include sudden onset, few prodromal symptoms, typical chest pain, fewer complications, low mortality, and a favorable prognosis. Regarding coronary artery lesions, single-vessel disease is predominant, while a certain proportion of patients present with normal coronary angiography results.	6
136	Zhou Qing	Chin.J.Integr.Med.Cardio-Cerebrovasc. Dis.	2012	Retrospective Case-Co	174	Male sex, alcohol consumption, smoking, and a family history are closely associated with acute myocardial infarction (AMI) in young patients. They typically present with characteristic chest pain that is often triggered by identifiable factors, and single-vessel disease is the predominant coronary artery lesion.	6
137	Li Zhao	Chin J Emerg Med	2012	Retrospective Case-Co	304	Obesity, type 2 diabetes mellitus, hypertension, a family history of premature coronary heart disease, smoking, and hypohemoglobinemia are the major risk factors for acute myocardial infarction (AMI) in young men.	6
138	Li Zhao	Chin J Cardiol	2012	Retrospective Case-Co	195	Essential hypertension, lower hemoglobin and uric acid are risk factors of young women with AMI. Single vessel coronary disease is the most common coronary angiographic feature of young women with AMI	6
139	Yang Ru	PJCCPVD	2012	Retrospective Case-Co	228	The risk factors for acute myocardial infarction (AMI) in young adults have their own distinct characteristics. Reducing unhealthy lifestyle habits is beneficial for lowering the incidence of AMI in this population.	7
140	Liu Xinyu	Journal of Shandong Medical College	2012	Retrospective Cohort S	162	Smoking cessation, lowering blood lipid levels, and standardized secondary prevention of coronary heart disease (CHD) in young adults help prevent the onset of AMI.	5

141	Liu Hao	Chinese General Practice	2012	Retrospective Cohort S	504	Smoking, alcohol consumption, a positive family history, and dyslipidemia predominantly characterized by elevated triglyceride (TG) levels are the primary risk factors for premature coronary heart disease (CHD) and acute myocardial infarction (AMI). The main clinical characteristics of patients in the premature group include male predominance and lower incidences of hypertension and diabetes mellitus	6
142	Ou Fengrong	Chinese Journal of Practical Internal Medicine	2012	Retrospective Case-Co	165	Smoking，mankind，abnormal blood lipid levels are important risk factors for premature coronary heart disease; increased plasma fibrinogen levels may serve as risk predictor for acute myocardial infarction of premature coronary heart disease．	5
143	Chen Qingqing	Guide of China Medicine	2012	Retrospective Case-Co	148	The occurrence of AMI in young adults is clearly associated with smoking, dyslipidemia, and a family history of premature cardiovascular disease. Quitting smoking and alcohol consumption, engaging in moderate physical exercise, and maintaining a balanced diet can reduce the incidence of AMI in this population.	6
144	Zheng Gaoshu	Chin J Postgrad Med	2012	Retrospective Case-Co	84	Young AMI patients exhibit a high proportion of males, heavy smoking and alcohol consumption, positive family history, and identifiable precipitating factors. They predominantly present with typical chest pain but lack a history of angina. Coronary angiography (CAG) primarily reveals single-vessel disease.	4
145	Lu Xiangsheng	Journal of Clinical Rational Drug Use	2012	Retrospective Case-Co	118	The proportions of smoking, excessive alcohol consumption, family history, obesity, and psychological stress among young and middle-aged AMI patients were significantly higher than those in the elderly group.	6

146	Hu Lihua	Chin J Cardiovasc Rehabil Med	2012	Retrospective Case-Cc	136	Compared with elderly patients with acute myocardial infarction (AMI), those with premature AMI were predominantly male and exhibited a significantly higher prevalence of smoking and family history of coronary heart disease, along with markedly elevated lipid levels. Furthermore, the premature AMI group had a higher proportion of single-vessel coronary artery disease and significantly less severe coronary stenosis.	6
147	Ma He	Diseasea monitor & control	2013	Retrospective Case-Cc	100	In young patients with acute myocardial infarction, the prominent risk factors are smoking and a family history of premature coronary artery disease. Common precipitating factors include strenuous exercise, emotional disturbances, and excessive fatigue. Clinically, these patients typically present with classic chest pain symptoms, while complications such as cardiac insufficiency, malignant arrhythmias, and cardiogenic shock occur less frequently compared to elderly patients. Coronary angiography often reveals single-vessel disease, with collateral circulation rarely observed.	6
148	Zhang Xiaojun	ournal of Hebei North University (Natural Science Edition)	2013	Retrospective Case-Cc	120	Compared with elderly AMI patients, the young group showed significant differences in risk factors such as a history of smoking, diabetes mellitus, and hypertension, as well as in the presentation of typical chest pain. Coronary angiography revealed that coronary artery lesions were predominantly single-vessel disease in the young group, whereas the proportion of multi-vessel disease was significantly higher in middle-aged and elderly patients.	6
149	Liu Dan	diseasea monitor & control	2013	Retrospective Cohort !	302	Smoking and alcohol consumption are the major influencing factors for coronary heart disease (CHD) in young patients.	6

150	Li Ruina	MEDICALLABORA TORY SCIENCES	2013	Retrospective Case-Co	186	The proportions of smoking and family history, as well as hemoglobin levels, TC, TG, LDL-C, BMI, and the proportion of single-vessel coronary artery disease, were significantly higher in the young group than in the middle-aged/elderly and advanced-age groups. Conversely, the proportions of hypertension and multi-vessel coronary artery disease, along with Gensini scores, were significantly lower in the young group compared to the other two groups.	5
151	Li Fujun	Chin. J. Cardiovasc. Res	2013	Cross-sectional Study	225	The prevalence of coronary heart disease (CHD) risk factors, such as smoking, dyslipidemia, hypertension, and diabetes mellitus, varies across male and female acute myocardial infarction (AMI) patients in different age groups. As age increases, multi-vessel and complex coronary artery lesions become more common; therefore, corresponding preventive and therapeutic measures should be implemented based on specific age groups.	6
152	Li Dan	Guide of China Medicine	2013	Retrospective Cohort S	136	Smoking, hyperlipidemia, and hypertension are significant risk factors for acute myocardial infarction (AMI) in young patients. Single-vessel disease is common in this group, and they have a favorable prognosis following percutaneous coronary intervention (PCI).	5
153	Ma YongBo	Nurs. Pract. Res.	2013	Retrospective Case-Co	388	It is essential to fully recognize the facilitating role of genetic factors in young AMI and remain vigilant about the impact of unhealthy lifestyles. Health education should begin in childhood, encouraging individuals to stay away from smoking, maintain a healthy weight, follow a balanced diet, and lead a regular lifestyle.	5

154	Hu XingXing	Chin J Evid Based Cardiovasc Med,	2013	Retrospective Case-Co	310	Compared with elderly patients, young AMI patients have a lower incidence of comorbidities but a higher prevalence of smoking. Furthermore, fewer coronary arteries are typically involved in young AMI patients compared to the elderly, with predominantly single-vessel disease being observed.	5
155	Liu Bing	Chin. J. Pract. Rural Doctor	2013	Retrospective Cohort S	168	Smoking, obesity, irregular lifestyle, and a family history of coronary heart disease (CHD) are significant risk factors for young patients with acute myocardial infarction (AMI). Their clinical manifestations and characteristics of coronary artery lesions differ markedly from those in elderly patients, resulting in a more favorable prognosis.	6
156	Xia Wei	Jilin Medical Journal	2013	Retrospective Case-Co	304	Smoking and family history of coronary heart disease are the major risk factors for AMI among the young pa tients. The young patients have milder coronary artery stenosis and lower mortality rate than elderly patients.	7
157	Li Zhao	J Chin Pract Diagn Ther	2013	Retrospective Case-Co	486	Metabolic syndrome is an independent risk factor for acute myocardial infarction (AMI) in young males (OR = 4.125) and is associated with multivessel coronary artery disease.	6
158	Li Zhao	Nat Med J China	2013	Retrospective Case-Co	623	HDL-C serves as a protective factor against young male AMI and multivessel coronary artery disease; smoking is associated with reduced HDL-C levels; while diabetes mellitus, hypertension, family history, smoking, and LDL-C constitute independent risk factors for AMI.	6
159	He Hankang	Chinese Journal of Integrative Medicine on Cardio/Cerebrovascular Disease	2013	Retrospective Case-Co	212	The young and middle-aged AMI cohort exhibited a significantly higher proportion of males and a greater prevalence of risk factors—including smoking, alcohol abuse, positive family history, and obesity—compared to the elderly group	6

160	Zeng Tongjun	Medical Consultation and Medication Inquiry (Academic Edition)	2013	Cross-sectional Study	126	Heavy smoking and dyslipidemia were the predominant contributing factors in young patients with acute myocardial infarction (AMI), who exhibited a favorable short-term prognosis	5
161	Riu Limei	Jiangsu Medical Journal	2013	Cross-sectional Study	55	Premature CHD-related MI was predominantly driven by traditional risk factors, specifically smoking, excessive alcohol consumption, dyslipidemia, hypertension, diabetes, and positive family history	4
162	Li Meilan	Guide of China Medicine	2013	Cross-sectional Study	36	Young AMI patients were predominantly male. Heavy smoking was the leading risk factor	4
163	Li Minfeng	Chin J Geriatr Cardiovasc Cerebrovasc Dis	2013	Retrospective Case-Co	84	Smoking constitutes the most critical risk factor for AMI in the young cohort. Psychologically, young AMI patients predominantly manifest anxiety, whereas elderly AMI patients are closely associated with hypertension and diabetes, with depression being their primary psychological presentation	6
164	Hu Bin	Journal of Cardiovascular & Pulmonary Diseases	2014	Retrospective Case-Co	296	The main risk factors for AMI patients aged ≤35 years are smoking and obesity. Most cases present as ST-segment elevation myocardial infarction (STEMI), with single-vessel coronary artery disease being the predominant lesion pattern.	6
165	Chang Lina	Chinese Journal of Integrative Medicine on Cardio-Cerebrovascular Disease	2014	Retrospective Cohort S	120	Smoking, hyperlipidemia, and hypertension are significant risk factors for acute myocardial infarction (AMI) in young patients. Single-vessel disease is commonly observed in this population, and the prognosis following percutaneous coronary intervention (PCI) is generally favorable.	6
166	Li Zhao	China Med. J.	2014	Retrospective Case-Co	64	Hypertension is an independent risk factor for acute myocardial infarction (AMI) in young women. Furthermore, AMI patients with concomitant hypertension present with more severe coronary artery lesions.	6

167	Cai Xiaojie	Prevention and Treatment of Cardio-Cerebra-l Vascular Disease	2014	Retrospective Case-Co	239	Smoking, hyperlipidemia, and male sex are the primary risk factors for acute myocardial infarction (AMI) in young patients, whereas elderly AMI patients frequently have comorbidities such as hypertension, diabetes mellitus, and chronic renal insufficiency. Killip class, the severity of coronary artery disease, and left ventricular systolic function remain significant prognostic factors.	6
168	Zhang Qian	Chin J Evid Based Cardiovasc Med	2014	Cross-sectional Study	114	Young patients with first-onset acute myocardial infarction (AMI) are predominantly male, often presenting with a distinct family history and identifiable precipitating factors. They typically have multiple high-risk factors, and coronary angiography mostly reveals single-vessel disease. Early monitoring and intervention may improve their prognosis.	6
169	Zhang Ying	Medical Journal of National Defense Force of China	2014	Retrospective Case-Co	546	Smoking, alcohol consumption, metabolic disorders, and a family history are the primary risk factors for acute myocardial infarction (AMI) in patients aged ≤ 40 years. The severity of coronary artery lesions is significantly less severe in young AMI patients compared to elderly patients.	5
170	Wang Ling	Medical Journal of Chinese People′s Health	2014	Retrospective Case-Co	112	The abdominal obesity，smoking，drinking，family his tory of premature coronary heart disease and dyslipidemia are the major risk factors for the young patients with acute STEMI． The caus ative factors of excessive fatigue， mood disorders， over eating and drinking are more frequently found among the young patients． Coro nary arteriography shows that the young patients show the higher incidence of one 一 vessel disease， mainly involving the proximal anteri or descending branch， and the lesions are in the low 一 risk group．	6

171	Yan Chaojin	J. Prim. Med. Forum	2014	Retrospective Case-Co	128	Smoking and a family history of coronary heart disease (CHD) are the primary risk factors for acute myocardial infarction (AMI) in young patients. Timely reperfusion therapy can significantly improve the prognosis of AMI patients.	5
172	Guo Yunfei	Inner Mongolia Med J	2014	Retrospective Case-Co	90	The young patient group is predominantly male. The main risk factors include smoking, obesity, high mental stress, heavy alcohol consumption, and a family history of coronary heart disease (CHD). Their clinical prognosis is generally favorable.	5
173	Lu Xin	Chinese Journal of Integrative Medicine on Cardio-Cerebrovascular Disease	2014	Retrospective Case-Co	95	The high-risk profile for young patients with acute myocardial infarction (AMI) is predominantly male, accompanied by precipitating factors such as heavy smoking, excessive alcohol consumption, extreme fatigue, high mental stress, and insomnia. Coronary angiography in these patients mostly reveals single-vessel disease.	6
174	Zhao RuiYang	Cardiovascular Disease Journal of integrated traditional Chinese and Western Medicine	2014	Retrospective Cohort S	100	Young patients with acute myocardial infarction (AMI) mainly present with typical chest pain and arrhythmias. Hyperlipidemia, hypertension, and smoking are the major precipitating factors leading to AMI in this population.	6
175	Yang JinLing	Clinical Focus	2014	Retrospective Case-Co	202	The primary risk factors for young patients with acute myocardial infarction (AMI) include male sex, smoking, alcohol consumption, a family history of premature coronary heart disease, hypertriglyceridemia (high TG), hyperuricemia (high UA), and low HDL-C. Fatigue and alcohol consumption are the main precipitating factors. These patients typically present with single-vessel and limited lesions, resulting in a low Gensini score, fewer complications, and low mortality.	7
176	Liu Ying	Chin J Cardiovasc Med	2014	Retrospective Cohort S	727	Smoking，obesity，early familial history of CHD and high TG were independent risk factors of AMI in men under the age of 40． The young male patients with AMI had better 1-year prognosis with PCI．	7

177	Tian Zhuang	Chin J Intervent Cardiol,	2014	Retrospective Case-Cc	94	Non-atherosclerotic factors should be considered in young women presenting with acute myocardial infarction (AMI). Compared to older female AMI patients, young women with atherosclerotic MI have fewer risk factors, and their coronary artery lesions are predominantly single-vessel.	5
178	Zhao Xianghai	Shandong Medical Journal	2014	Retrospective Cohort S	150	Young patients with acute myocardial infarction (AMI) have well-defined risk factors, including smoking, alcohol consumption, hypertension, hyperlipidemia, and genetic factors. Emphasizing the control of these risk factors, enforcing strict smoking cessation, strengthening prevention and management, and implementing early preventive measures for high-risk individuals with a genetic predisposition can significantly reduce the incidence of AMI.	6
179	Zhang Yong	Chin J Cardiovasc Rehabil	2014	Retrospective Case-Cc	401	Smoking, obesity, and hyperlipidemia are the most significant risk factors for acute myocardial infarction (AMI) in patients under 30 years of age. Staying up late, alcohol consumption, and high psychological stress serve as the primary precipitating factors for AMI in young adults.	7
180	Yu Aixiang	Medical Information	2014	Retrospective Case-Cc	60	Obesity, hypertension, hyperlipidemia, hyperglycemia, a family history of the disease, and unhealthy lifestyle habits are precipitating factors for acute myocardial infarction (AMI) in middle-aged and young adults.	6

181	Wang Qihu	Journal of Frontiers of Medicine	2014	Retrospective Case-Cc	95	Young patients with acute myocardial infarction (AMI) are associated with multiple risk factors. The predominant coronary artery lesion characteristics include left anterior descending (LAD) artery lesions, right coronary artery (RCA) lesions, and thrombosis formation. It is essential to proactively control and reduce unhealthy lifestyle habits, undergo regular physical examinations, and implement early preventive measures against adverse coronary events.	5
182	Li Zhao	China Medicine	2014	Retrospective Case-Cc	124	Hypertension is an independent risk factor for AMI in young women. Furthermore, AMI patients with comorbid hypertension exhibit more severe coronary artery lesions.	8
183	Yang Ming	Chin J Intervent Cardiol	2014	Retrospective analysis	1204	In AMI patients aged ≤30 years, ST-segment elevation myocardial infarction (STEMI) is the predominant presentation. Single-vessel disease is commonly observed, with the left anterior descending (LAD) artery being the most frequent culprit vessel. Female patients frequently have concurrent autoimmune diseases, with Takayasu arteritis being the most common. Short-term prognosis following percutaneous coronary intervention (PCI) is favorable, whereas long-term prognosis depends on underlying comorbidities.	7
184	Ma Jing	Guide of China Medicine	2014	Retrospective Case-Cc	336	Young AMI patients are predominantly male, with high rates of smoking and alcohol consumption, but a low prevalence of hypertension and diabetes mellitus. Coronary artery lesions are mainly single-vessel disease involving the left anterior descending (LAD) artery, and the proportion of normal coronary angiography (CAG) findings is higher than that observed in elderly patients	6

185	Wu Jia	Contemporary Medicine	2014	Retrospective Case-Cc	140	Young and middle-aged AMI patients are predominantly male, with a higher prevalence of smoking, alcohol consumption, obesity, and positive family history compared to the elderly. In contrast, the elderly group exhibits a higher prevalence of hyperglycemia and dyslipidemi	6
186	Peng Jiren	Zhejiang Clinical Medical Journal	2014	Retrospective Case-Cc	58	Young male AMI patients are characterized by a high prevalence of heavy smoking (82.8%), positive family history (44.8%), and identifiable precipitating factors (79.3%). They predominantly present with typical chest pain, and coronary angiography (CAG) primarily reveals single-vessel disease involving the left anterior descending artery (LAD) (79.3%), leading to a favorable prognosis following percutaneous coronary intervention (PCI).	6
187	Zhao Nishan	World Latest Medicine Information	2014	Retrospective Case-Cc	155	The smoking rate was significantly higher in the middle-aged and young AMI group (76.5%) compared to the elderly group (35.6%). Conversely, the prevalence of hypertension was significantly higher in the elderly group (64.4%) than in the middle-aged and young patients (41.2%); however, there was no significant difference in the cumulative burden of risk factors between the two groups.	6
188	Lu Zhaohua	Hainan Med J	2014	Retrospective Case-Cc	68	Smoking is an independent risk factor for acute myocardial infarction (AMI) in young adults.	6
189	Sun Li	Journal of Preventive Medicine Information	2014	Retrospective Case-Cc	200	Smoking, hypertension, hyperlipidemia, diabetes, and family history are independent risk factors for AMI; The smoking rate in young and middle-aged patients was significantly higher than in elderly patients; Controlling risk factors is of great significance for the primary prevention of AMI.	6

190	Chen Qi	Journal of Electrocardiology and Circulation	2015	Retrospective Cohort S	1354	Young patients with acute STEMI differ significantly from middle-aged and elderly patients in terms of risk factors, clinical presentations, coronary angiographic characteristics, and prognosis. Therefore, treatment strategies should be tailored to these specific features.	6
191	Zeng Jun	Medical Information	2015	Retrospective Case-Co	54	The primary risk factors for acute myocardial infarction (AMI) in young individuals, in descending order of importance, are smoking and a positive family history of hyperlipidemia. Overcoming adverse lifestyle habits is beneficial in reducing the incidence of AMI.	4
192	Bian Ling	Anhui Medical and Pharmaceutical Journal	2015	Cross-sectional Study	250	cardiovascular disease (CVD), and elevated low-density lipoprotein cholesterol (LDL-C). In contrast, diabetes mellitus is a significant risk factor for AMI occurring in middle-aged and elderly individuals.	5
193	Wang Hui	Chin J Intervent Cardiol	2015	Retrospective Cohort S	957	Among STEMI patients undergoing primary PCI, young patients were predominantly male compared with middle-aged and elderly patients. Smoking and hyperlipidemia were their main risk factors. They had fewer comorbidities, predominantly single-vessel coronary artery disease, more adequate pharmacological therapy, more timely reperfusion therapy, fewer complications, and better 30-day prognosis.	7
194	Yan Ning	Journal of China Medical University	2015	Retrospective Cohort S	189	Compared with young Han patients with acute myocardial infarction (AMI), young Hui patients tend to develop the disease at a relatively younger age, exhibit a higher prevalence of diabetes mellitus and three-vessel disease, but have lower rates of smoking and alcohol consumption	6

195	Huang Yu	World Latest Medicine Information(Electronic Version)	2015	Retrospective Case-Cc	172	Young and middle-to-elderly patients with acute myocardial infarction (AMI) have distinct clinical features. Once young individuals experience persistent chest pain, prompt evaluation with electrocardiogram (ECG) and cardiac enzyme testing should be performed, alongside strengthened prevention and control of various risk factors	5
196	Xu Yonggang	China Prescription Drugs	2015	Retrospective Case-Cc	80	Young patients with acute myocardial infarction (AMI) have distinct clinical characteristics. Young people should maintain a healthy lifestyle to prevent the onset of AMI.	6
197	Li BoYan	World Latest Medicine Information Abstracts	2015	Retrospective Case-Cc	612	The primary risk factors for myocardial infarction in young patients are smoking and hyperlipidemia. Risk factors should be actively controlled to prevent the progression of the disease. Since coronary lesions in this population are predominantly single-vessel, early revascularization is recommended to improve prognosis.	6
198	Xu Zhuo	International Journal of Cardiovascular Disease	2015	Retrospective Cohort S	624	Young patients with acute myocardial infarction (AMI) are predominantly male and present with risk factors such as smoking and dyslipidemia, along with a genetic predisposition. Acute ST-elevation myocardial infarction (STEMI) involving a single vessel is more common in this population, while complications related to cardiac insufficiency are relatively rare.	7
199	Wang GuiRong	HEILONGJIANG MEDICAL JOURNAL	2015	Retrospective Case-Cc	113	Smoking，drinking，tired，family history，fatness and hyperlipidemia are the important risk factors in young AMI patients. Compared with aging AMI patients， the symptom of young patients were typical and most cases attacked for the first time and presented STEMI. Most of their ves sel diseases were single vessel.	5

200	Zhang Bin	Hianan Med	2015	Retrospective Case-Cc	665	HDL-C is a protective factor against acute myocardial infarction (AMI) and multi-vessel coronary artery disease in young women, and can serve as an important clinical monitoring indicator for AMI prevention	6
201	Zhou Jiangrong	Anhui Medical Journal	2015	Retrospective Cohort S	225	Young and elderly patients with AMI differ not only in risk factors and clinical manifestations, but also show significant differences in the characteristics of coronary artery lesions. Young patients with AMI are frequently associated with risk factors such as smoking, hyperlipidemia, obesity, and a family history of coronary heart disease (CHD).	7
202	Zhang Xianhua	Medical Innovation of China	2015	Retrospective Cohort S	444	Young patients with AMI typically present with classic chest pain and involvement of fewer coronary arteries. For males with a positive family history, addressing unhealthy habits can help reduce the risk of developing the disease.	6
203	Feng Tianlong	Guide of China Medicine	2015	Cross-sectional Study	124	A family history of coronary heart disease (CHD), a history of smoking, and hypercholesterolemia are the primary risk factors for CHD in middle-aged and young patients, whereas hypertension and diabetes mellitus are the predominant risk factors in the elderly. Among middle-aged and young adults, particularly those with genetic predispositions, controlling smoking and lipid levels is key to preventing CHD.	5
204	Geng Qian	Acta Cardiol Sin	2015	Retrospective Cohort S	9462	We demonstrated that cigarette smoking is the most prevalent factor among the avoidable cardiovascular risk factors for young ST-segment elevation myocardial infarctions in China. Accordingly, continued smoking is the most powerful predictor for the recurrence of cardiac events in young Chinese patients with ST-segment elevation myocardial infarction.	6

205	Gu Yunfei	J Clin Cardiol (China)	2015	Retrospective Case-Co	570	Serum cystatin C (CysC) levels are significantly elevated in the young STEMI group compared to the control group, and CysC is identified as an independent risk factor for young STEMI (OR = 2.408).	6
206	Tang Xiaolan	Chin J Public Health Adm	2015	Retrospective Case-Co	150	Young AMI patients exhibit a higher prevalence of smoking and positive family history compared to the elderly. There is marked clustering of precipitating factors (such as overwork and heavy smoking), and the concomitance of multiple precipitating factors readily predisposes young individuals to acute myocardial infarction (AMI).	6
207	Yan Cuiping	China Modern Doctor	2015	Retrospective Case-Co	78	Smoking, a family history of premature coronary artery disease, and hypertriglyceridemia are independent risk factors for young AMI. Coronary angiography predominantly reveals single-vessel disease (55.3%) with predominant involvement of the left anterior descending artery (LAD), and the left ventricular ejection fraction (LVEF) is higher than that in middle-aged and elderly patients.	6
208	Huang Jian	Jiangsu Med J	2015	Retrospective Case-Co	247	A family history of premature coronary artery disease, overweight, unhealthy lifestyles, and elevated total cholesterol (TC) or low-density lipoprotein (LDL) are pivotal factors for young male AMI. Coronary angiography predominantly reveals single-vessel disease with a low Gensini score.	6
209	Li Dan	China Prac Med	2015	Retrospective Case-Co	166	The young female AMI group exhibits a higher prevalence of hypertension, diabetes mellitus, smoking, and elevated LDL-C compared to elderly female AMI patients. Coronary artery involvement predominantly affects the left anterior descending artery (LAD), with single-vessel disease being more common.	6

210	Zhao Hongtao	China Modern Doctor	2015	Retrospective Case-Co	150	Young AMI patients are predominantly male, with smoking, alcohol consumption, and high work stress being the primary risk factors. Chest pain is the main symptom, and ST-segment elevation is frequently observed on electrocardiograms (ECG). Coronary angiography typically reveals single-vessel disease, most commonly involving the left anterior descending (LAD) artery. Timely reperfusion therapy leads to a favorable prognosis.	5
211	Wang LiFang	Journal of Chinese Physician	2015	Retrospective Case-Co	126	The proportions of hypertension, diabetes, and family history were higher in young female AMI patients compared to young males; There was a higher proportion of multi-vessel coronary artery disease; A history of oral contraceptive use was associated with an increased risk of AMI; Special risk factors for young females require attention.	6
212	Yang Ze	Chinese Journal of Health Laboratory Technology	2015	Retrospective Case-Co	564	Middle-aged and elderly patients predominantly exhibited multi-vessel disease, mostly representing an acute exacerbation of chronic lesions. In contrast, coronary involvement in young MI patients was primarily confined to the left anterior descending (LAD) artery, with relatively singular lesions. This pattern may be attributed to greater hemodynamic shear stress on the vessels and suggests a significant role for genetic factors.	6

213	Hao Wen	Chinese Circulation Journal	2016	Retrospective Cohort S	685	Among young AMI patients under 40 years of age, males are predominant. The average age of male patients is higher than that of females. Female patients have a higher proportion of left main coronary artery lesions and are more prone to acute heart failure during hospitalization. Elevated levels of high-sensitivity troponin I and multi-vessel coronary artery disease increase the incidence of major adverse cardiovascular events (MACE) in young male patients, whereas percutaneous coronary intervention (PCI) is associated with favorable prognosis in male patients.	6
214	Lu Zongquan	Chinese Journal of Clinical Medicine	2016	Retrospective Case-Co	120	Smoking and a family history of premature coronary artery disease are the main pathogenic factors for acute myocardial infarction in young patients under 40 years of age, with single-vessel lesions being predominant.	5
215	Yan Rongli	China & Foreign Medical Treatment	2016	Retrospective Case-Co	80	Compared with younger patients (under 40 years of age), elderly patients with acute myocardial infarction present with atypical clinical symptoms, and exhibit higher morbidity and mortality rates.	5
216	Xie Wei	Journal of Bethune Medical Science	2016	Retrospective Cohort S	144	The young AMI group had a lower prevalence of diabetes mellitus, hypertension, and hypertriglyceridemia compared with the elderly group, but a higher prevalence of a family history of AMI. The incidence of extensive anterior wall, multi-site, and true posterior wall myocardial infarction was lower in the young group than in the elderly group. Furthermore, both the complication rate and mortality were significantly lower in the young group.	6

217	Shi TingTing	Journal of Qiqihar University of Medicine	2016	Retrospective Case-Cc	112	The proportions of smoking and a family history of cardiovascular disease are significantly higher in the young group than in the elderly group, whereas the rates of diabetes and hypertension are significantly lower. The risk factors for AMI differ between young patients and the elderly, leading to different priorities in prevention and management. Young people should adopt early preventive measures and correct unhealthy lifestyle habits, which is conducive to reducing the incidence of AMI	5
218	Liu Yan	Medical Journal of Chinese People′s Health	2016	Retrospective Cohort S	196	The incidence of acute myocardial infarction (AMI) in young patients is on the rise year by year. Improving lifestyle, controlling blood pressure, and smoking cessation are effective measures.	6
219	Liu FuRong	Chin J Clinicians(Electronic Edition)	2016	Retrospective Cohort S	183	The clinical characteristics and related conditions of acute myocardial infarction (AMI) in young patients differ significantly from those in the elderly. Differences are observed in the site of infarction, cardiac function, and the incidence of adverse events. The prognosis for young patients is more favorable than that for elderly patients; therefore, tailored preventive measures for coronary heart disease should be implemented for different populations.	6
220	Jin Jian	China Prac Med	2016	Retrospective Cohort S	194	The primary risk factors for young patients with acute myocardial infarction (AMI) are male sex, smoking, and hyperlipidemia. Their coronary artery lesions are predominantly single-vessel disease with a heavy thrombus burden, frequently involving the left anterior descending (LAD) artery. The short-term prognosis is relatively favorable.	7

221	Ke ZhiQuan	Journal of Luzhou Medical College	2016	Retrospective Case-Co	748	Smoking, obesity, high blood pressure, diabetes, high TG and LDL-C were independent risk factors for AMI in men under the age of 45. The young patients with AMI tended to have a favorable prognosis in long term compared with old patients, so young male patients should be given a PCI as early as possible.	6
222	Yin Hong	Guide of China Medicine	2016	Retrospective Cohort S	111	The primary risk factors leading to acute myocardial infarction (AMI) in young adults include smoking, irregular lifestyles, obesity, dyslipidemia, and a family history of the disease. Clinically, management focuses primarily on patient education, prevention, and lifestyle modifications	6
223	Chen Minghui	Chinese and Foreign Medical Research	2016	Retrospective Cohort S	76	Young patients with acute ST-segment elevation myocardial infarction (STEMI) often present with risk factors such as smoking and being overweight, and their coronary artery disease is predominantly characterized by single-vessel lesions. In contrast, elderly patients with acute STEMI are frequently concomitant with diabetes mellitus and hypertension, and their coronary angiography reveals predominantly multi-vessel lesions.	6
224	Chu Juan	World Latest Medicine Information (Electronic Version) 2	2016	Cross-sectional Study	98	Dyslipidemia is the primary risk factor for patients with premature coronary heart disease (CHD) and myocardial infarction. These patients are predominantly male and typically present with predominantly single-vessel lesions, while exhibiting relatively lower rates of hypertension and diabetes mellitus. Furthermore, this population is associated with a higher mortality rate.	4

225	Lei Yan	Cardiovascular Disease Journal of integrated traditional Chinese and Western Medicine	2016	Cross-sectional Study	120	The risk factors for premature coronary heart disease (CHD) and myocardial infarction (AMI) include a history of smoking, elevated triglyceride (TG) levels, a positive family history, and alcohol consumption. Clinically, this condition is characterized by a male predominance and relatively lower incidences of hypertension and diabetes mellitus.	4
226	Gao Xiaojin	Natl Med J china	2016	Prospective Cohort St	24379	there were different factors for AMI patients in different age groups, and young AMI patients were predisposed to live an unhealthy lifestyle	7
227	Wang Ping	Journal of Medical Research	2016	Retrospective Cohort	814	Male， smoking， hypertriglyceridemia may be risk factors of young and middle aged AMI.	8
228	Yang Cheng	World Latest Med Info	2016	Retrospective Case-C	80	Young AMI patients are predominantly male, with high prevalence rates of smoking and alcohol consumption, and primarily present with single-vessel coronary artery disease. In contrast, middle-aged and elderly AMI patients exhibit a higher prevalence of hypertension and diabetes mellitus, and are characterized by multivessel disease.	6
229	Han Quanle	Chinese Circulation Journal	2016	Prospective Cohort St	62208	Age, male sex, LDL-C, and TG are risk factors for AMI in young and middle-aged populations.	7
230	Cao Na	Xuzhou Med Univ	2017	Retrospective Cohort	266	LDL-C, TG, hypertension, and BMI are the primary risk factors for non-smoking young and middle-aged patients with acute myocardial infarction (AMI). Furthermore, single-vessel coronary artery disease is predominantly observed in this specific patient population.	8
231	Zhao Maosun	The Journal of Primary Medical Forum	2017	Retrospective Case-C	95	There are significant differences in risk factors between young and elderly patients with acute myocardial infarction (AMI). The proportion of males, as well as the prevalence of smoking, alcohol consumption, and a family history of AMI, are significantly higher in the young group than in the elderly group.	4

232	Su Dayu	China &Foreign Medical Treatmen	2017	Retrospective Cohort !	120	The heavy smok ing, obesity, hyperlipemia and coronary heart disease family history are the major risk factors of young patients with myocar dial infarction, and we should widely carry out the health education, advocate the healthy life of style of young people in cluding law of life, quitting smoking, controlling weight, monitoring blood pressure, blood sugar and blood fat, and avoiding the overwork and mood swings can the major measures of preventing the occurrence of young STEMI, and the young people should see a doctors as soon as possible once the disease has started and actively adopt the coronary interventional treat ment thus improving the prognosis.	6
233	Li Zhao	China Medicine	2017	Retrospective observa	1032	The number of young patients with acute myocardial infarction (AMI) is on the rise, while the proportion of single-vessel disease shows a downward trend. Meanwhile, the prevalence of dyslipidemia has been increasing year by year	6
234	Zhang Ruqiang	Journal of Kunming Medical University	2017	Retrospective Case-Co	850	The risk factors of CAD / MI are closely related to the age and gender of the patients． and the preventive measures should be taken according to the age and gender to reduce the incidence of CAD / MI．	5
235	Zhao Ning	Chinese Journal of Cardio v ascular Research	2017	Retrospective Case-Co	129	The patients with early myocardial infarction is mainly with single vessel disease， LDL-C， smo k ing history and family history are the independent ris k factors for early myocar dial infarction； the patients with early myocardial infarction have low mortality and favorable prognosis.	6
236	Zhang Jiayun	China Medicine	2017	Retrospective Case-Co	200	Elevated heart rate, a history of diabetes mellitus, and high serum uric acid levels are independent risk factors for acute ST-segment elevation myocardial infarction (STEMI) in middle-aged and young women. In contrast, elevated HDL-C levels serve as an independent protective factor	6

237	Lu Xiuying	Journal of Ningxia Medical University	2017	Retrospective Cohort S	158	Compared with the elderly AMI group, young patients exhibit a higher prevalence of smoking history, alcohol consumption history, and a family history of coronary artery disease. Young AMI is frequently associated with distinct precipitating factors, typical chest pain episodes, and arrhythmias.	6
238	Liu Zongwu	China Health Industry	2017	Cross-sectional Study	266	The prevalence of smoking, alcohol consumption, and obesity is significantly higher in the young group compared to the elderly group, whereas the elderly group exhibits higher levels of blood glucose and blood lipids.Given the distinct distribution patterns of risk factors across different age groups, risk-stratified interventions are essential.	6
239	Zhai Gongwei	Medical Information	2018	Retrospective Case-Co	51	Male sex, smoking, concomitant hypertension, and concomitant hyperlipidemia are prominent risk factors for STEMI patients under 30 years of age. Their coronary artery lesions are predominantly single-vessel and type A, with favorable clinical outcomes within 30 days of onset	4
240	Zhang Zhili	Chin J Emerg Resusc Disaster Med，	2018	Retrospective Cohort S	158	High body mass index (BMI), hyperlipidemia, and smoking are the primary risk factors for young patients.	6
241	Gu Ning	Diet & Health Care	2018	Retrospective Cohort S	2414	The incidence of acute myocardial infarction (AMI) is shifting toward a younger demographic. This trend may be attributed to the high prevalence of diabetes mellitus, hypertension, and dyslipidemia among young and middle-aged patients in recent years, which is largely driven by their unhealthy lifestyles.	7
242	Sun Yanxiang	J. Pract. Med.	2018	Retrospective Case-Co	276	Male sex, a history of hypertension, elevated triglycerides (TG), and mean platelet volume (MPV) are the major risk factors for premature coronary heart disease (CHD) presenting with acute myocardial infarction (AMI).	7

243	Wu ZhenYu	Journal of Clinical Internal Medicine	2018	Retrospective Cohort S	129	Young patients with acute myocardial infarction (AMI) are predominantly male. Overweight or obesity, hyperlipidemia, and a family history of coronary heart disease (CHD) are common contributing factors. This group has a higher likelihood of presenting with typical chest pain, and their coronary artery lesions mainly manifest as single-vessel infarction. Furthermore, the in-hospital incidence of major adverse cardiovascular events (MACE) is relatively low.	6
244	Yang FeiYun	Journal of Xinxiang Medical University	2018	Retrospective Case-Co	536	In young patients with acute myocardial infarction (AMI), coronary artery lesions predominantly involve single-vessel disease, most frequently affecting the left anterior descending (LAD) artery. Given the multitude of risk factors contributing to AMI in this population, targeted preventive measures should be implemented for each specific factor to reduce the incidence of AMI among young individuals.	7
245	Liu Hongcui	China & Foreign Medical Treatment	2018	Retrospective Cohort S	108	There are distinct clinical differences between young and elderly patients with myocardial infarction (MI). In young patients, male sex, a positive family history, and dyslipidemia are the primary risk factors, and they typically present with classic chest pain at onset. Furthermore, young patients exhibit more favorable outcomes regarding the degree of stenosis and the number of affected coronary arteries compared to their elderly counterparts, resulting in an overall better prognosis.	6
246	Zhu Xiaojian	Clinical Research	2018	Cross-sectional Study	76	Smoking was a major risk factor for acute myocardial infarction in young patients under 40 years of age who had a special pattern of onset． Understanding these characteristics could provide the basis for clinical prevention and treatment．	4

247	Zhang Huiying	JOURNAL OF HEZE MEDICAL COLLEGE	2018	Retrospective Case-Cc	100	Acute myocardial infarction (AMI) predominantly affects young males, with smoking, alcohol consumption, and obesity being the most prominent risk factors. Coronary artery involvement is mostly characterized by single-vessel disease, and patients typically present with classic chest pain symptoms.	5
248	Tian Tingting	Hindawi	2018	Retrospective Case-Cc	2210	increased serum UA levels were associated with EOCAD and first indicated that serum UA is an independent risk factor for the disease. We also demonstrated that the serum UA levels were associated with disease severity, affected with drinking status, and were pos itively correlated with SCr and weight. The findings may be useful in understanding the mechanism and predicting pro gression of patients with EOCAD and may contribute to the further prevention of the disease.	8
249	Maiwulajiang Mamuti	China Health Care & Nutrition	2018	Retrospective Case-Cc	212	Family history, obesity, hyperlipidemia, hypertension, and diabetes are established risk factors for young and middle-aged AMI patients, whereas smoking and excessive alcohol consumption act as precipitating factors.	6
250	Zhan Xiaona	Chinese Journal of Current Medical Sciences	2019	Retrospective Cohort S	315	Compared with the middle-aged and elderly group, young STEMI patients exhibit significantly higher proportions of heavy smoking, staying up late, high psychological stress, being single, overwork, family history, hyperlipidemia, hyperuricemia, and high body mass index (BMI). Conversely, they have lower rates of hypertension and diabetes mellitus, along with a higher prevalence of single-vessel disease.	6
251	Yang Yanmei	Mod. J. Integr. Tradit. Chin. West. Med.	2019	Cross-sectional Study	1740	Smoking, elevated LDL-C, and a family history of coronary heart disease (CHD) are the primary risk factors for young patients with acute myocardial infarction (AMI).	6

252	Sun Yunjuan	China Medicine	2019	Retrospective Case-Co	129	A history of diabetes mellitus, a history of stroke, and elevated apolipoprotein A1 are the primary risk factors for female patients with premature acute myocardial infarction (AMI). In contrast, a history of smoking, elevated homocysteine, and elevated low-density lipoprotein cholesterol (LDL-C) are the major risk factors for male patients. Furthermore, female patients exhibit a higher proportion of three-vessel disease and diffuse lesions compared to their male counterparts	8
253	Wang Yunchun	World Latest Medicine Information Abstracts	2019	Retrospective Cohort S	196	The prevalence of male sex, smoking history, and alcohol consumption was significantly higher in the young group than in the elderly group. In contrast, a history of hypertension and diabetes mellitus was significantly more common in the elderly group. No significant differences were observed between the two groups regarding family history of cardiovascular disease (CVD), overweight, and dyslipidemia. Most patients in the young group exhibited clinical manifestations or precipitating factors prior to onset, including irritability, impatience, emotional stress, excessive physical exertion, and chest pain.	6
254	E Jihong	Tibet Science and Technology	2019	Retrospective Cohort S	156	Young patients are characterized by smoking, alcohol consumption, hyperlipidemia, and emotional agitation, whereas elderly patients typically present with hypertension, hyperlipidemia, and smoking. Chest pain is the predominant initial symptom in non-middle-aged STEMI patients. Furthermore, elderly patients often have a more severe clinical presentation compared to their younger counterparts, predominantly involving multi-vessel coronary arteries.	6

255	Zheng Yan	Pract Prey Med	2019	Retrospective Case-Cc	393	Male sex, hypertension, hyperlipidemia, a family history of coronary heart disease (CHD), smoking, heavy alcohol consumption, and daily sleep duration <6 hours are associated risk factors for myocardial infarction among young and middle-aged adults in Wuhan. Therefore, it is recommended that this population quit smoking, limit alcohol intake, modify unhealthy lifestyle habits, prioritize regular medical check-ups, and detect risk factors such as hypertension and hyperlipidemia at an early stage to implement targeted preventive measures.	7
256	Zhang Jing	Journal of Aerospace Medicine	2019	Cross-sectional Study	821	Alcohol consumption, smoking, overweight, hypertension, a positive family history, and elevated triglyceride (TG) levels are closely associated with acute myocardial infarction (AMI) in premature coronary heart disease (CHD).	7
257	Sun Jing	Chinese Remedies & Clinics	2019	Retrospective Case-Cc	540	Males account for 95.2% of early-onset AMI cases, with a high prevalence of smoking (82.6%) and multivessel disease (59.5%). Compared to the control group, there are no significant differences in traditional risk factors (blood pressure, blood glucose, lipid profile, and BMI), suggesting a potential genetic predisposition. Anterior wall ST-segment elevation myocardial infarction (STEMI) is the most common presentation.	6
258	Zhu Zhe	Chinese Journal of Health Engineering	2019	Retrospective Case-Cc	252	The early-onset group had high proportions of males, overweight, hyperlipidemia, alcohol consumption, smoking, and family history, but lower rates of hypertension and diabetes, with high TG levels; More involvement of the left anterior descending artery and single-vessel disease; Smoking, family history, TG, hypertension, and diabetes were independent influencing factors.	6

259	Sun Jing	Chinese Remedies & Clinics	2019	Retrospective Case-Co	540	For young and middle-aged individuals with a positive family history of coronary heart disease, heavy smoking, and excessive alcohol consumption, regular health check-ups should be conducted. It is recommended to monitor blood pressure, regulate lipid profiles, quit smoking, limit alcohol intake, maintain psychological balance, and emphasize strengthening primary prevention for coronary heart disease	6
260	Hu Long	Journal of Electrocardiology and Circulation	2020	Retrospective Cohort S	1328	Smoking, high body mass index (BMI), and elevated low-density lipoprotein cholesterol (LDL-C) are the primary risk factors for acute myocardial infarction (AMI) in young patients.	7
261	Fu Li	China Medical Journal	2020	Retrospective Case-Co	494	Young and middle-aged patients should receive more aggressive interventions for smoking cessation, body weight control, and the management of triglyceride and serum uric acid levels. Furthermore, heightened vigilance is warranted for abnormally elevated hemoglobin. In contrast, elderly patients require comprehensive management to ensure that lipid profiles, blood glucose, blood pressure, and serum uric acid levels reach target goals.	7
262	Chen Houliang	Journal of Logistics Uninversity of PAP	2020	Retrospective Cohort S	71	For young patients with acute ST-segment elevation myocardial infarction (STEMI), smoking is the predominant risk factor affecting long-term prognosis.	5
263	Sun Chengcheng	Chin J Intervent Cardiol	2020	Retrospective Case-Co	174	The prevalence of chronic kidney disease (CKD) is high among women who experience myocardial infarction before menopause. CKD is a significant risk factor for coronary heart disease (CHD). Therefore, CHD prevention warrants particular attention in young and middle-aged female patients with CKD.	5

264	Niu Wenhao	Chin J Diffic and Compl Cas	2020	Retrospective Cohort !	380	Male sex and smoking are the primary risk factors for young patients with myocardial infarction (MI), who are susceptible to acute myocardial infarction (AMI) in the morning.	6
265	Li Zhanlu	Zhejiang Clinical Medical Journal	2020	Retrospective Cohort !	402	Smoking and a family history of premature coronary heart disease are risk factors for the onset of AMI in young patients, whereas the proportions of diabetes and hypertension are significantly higher in the elderly group. The survival rates of young patients during hospitalization, at 6 months post-procedure, and during long-term follow-up [mean (38.6 ± 20.5) months] were all higher than those of the elderly group.	7
266	Du Yong	Mod Diagn Treat	2020	Retrospective Case-Cc	160	The primary risk factors for acute myocardial infarction (AMI) in young patients include smoking, alcohol consumption, and a family history. Typical chest pain and anterior wall infarction are the main clinical characteristics of this condition.	7
267	Zhu Yeran	Health Care Guide	2020	Retrospective Case-Cc	200	The primary risk factors for acute myocardial infarction (AMI) in young and middle-aged adults include male sex, age, triglyceride levels, and low-density lipoprotein (LDL) levels. Monitoring these parameters plays a crucial role in controlling the progression of the disease.	7
268	Zhang Shaoyan	Bioscience Reports	2020	Retrospective Case-Cc	2630	Our findings indicated that G/A variant (rs6748040) and glutamic acid repeat polymorphism of the ALMS 1 gene associated with the risk of early-onset MI in the Chinese population.	8
269	Zhao Shenghuan	Journal of Frontiers of Medicine	2020	Retrospective analysis	29	The primary risk factors for AMI in young adults include male gender, smoking, and a family history of coronary heart disease. Coronary artery lesions are predominantly single-vessel disease, necessitating targeted prevention and treatment strategies.	4

270	Liu Miao	Journal of Jinzhou Medical University	2020	Retrospective Case-Cc	466	The most common risk factors for premature STEMI in males were smoking and hyperlipidemia, while in females they were hypertension and diabetes; Females had more atypical chest pain and delayed medical consultation, with higher in-hospital MACE and new-onset heart failure rates than males; Female sex itself was not an independent risk factor for MACE; Diabetes and delayed consultation time were independent risk factors for MACE.	7
271	Wang Xiao	Journal of Clinical Cardiology	2020	Cross-sectional Study	512	Young patients with STEMI were characterized by high rates of smoking and insufficient sleep. Compared to middle-aged and elderly individuals, they exhibited a higher prevalence of dyslipidemia and elevated LDL-C levels, alongside greater proportions of first-time diagnoses of hypertension and diabetes.	5
272	Tian Chunxiu	Chinese Remedies & Clinics	2020	Retrospective Case-Cc	243	Myocardial infarction in young adults tended to involve single-vessel coronary artery disease, with a predominance of male patients. Smoking and dyslipidemia were identified as the primary risk factors	6
273	Yue BoCheng	Chongqing Medicine	2020	Prospective Cohort Stı	54797	Age, male sex, BMI, SBP, FBG, TC, smoking, and fatty liver are risk factors for AMI in young and middle-aged populations, whereas alcohol consumption serves as a protective factor.	8

274	Li Xiangru1	Chin J Cardiol	2021	Retrospective Cohort :	2444	Among STEMI patients aged 18 to 35 years, the majority are male, obese, and smokers. The prevalence of hypertension and diabetes is relatively low, whereas levels of LDL-C, uric acid, and homocysteine are elevated. Coronary artery stenosis is common in this population, with lesions predominantly affecting a single vessel, particularly the left anterior descending (LAD) artery. Patients with normal coronary angiography have better prognoses than those with stenosis, while diabetes and dyslipidemia increase the risk of recurrent cardiovascular events	8
275	Tang XueHong	Knowledge of Cardiovascular Disease Prevention and Treatment	2021	Retrospective Cohort :	270	Young patients with acute myocardial infarction (AMI) are predominantly male. The primary risk factors include heavy smoking, a family history of coronary heart disease, excessive alcohol consumption, and obesity. Most patients present with typical angina symptoms, and single-vessel disease is predominant.	7
276	Che Yihong	Chinese Journal of Thrombosis and Hemostasis	2021	Retrospective Cohort :	162	The risk factors for acute myocardial infarction (AMI) include hypertension, diabetes mellitus, a history of alcohol consumption and smoking, a family history of coronary heart disease (CHD), and triglyceride (TG) levels. Furthermore, the patients predominantly present with single-vessel lesions.	6
277	Sun Zhaojin	Modem Medical Joumal	2021	Retrospective Cohort :	270	A family history of premature coronary heart disease, hypertriglyceridemia, and a history of smoking may serve as risk factors for premature myocardial infarction (PMI) in this region. Coronary artery lesions in PMI patients are predominantly single- or double-vessel lesions, making these patients more likely to undergo emergency revascularization.	7

278	Zuo Huijuan	Chin J Cardiol	2021	Retrospective Cohort S	283761	Smoking, dyslipidemia, and hypertension are most prevalent in China and United State. Significant sex and AMI subtype difference are observed for individual risk factors. The prevalence of hypertension and obesity increased significantly over time in China and all these five conventional risk factors increased significantly in the United States.	7
279	Zhang Min	Natl Med J China	2021	Cross-sectional Study	26592	There are gender differences in cardiovascular risk factors among Chinese patients with acute myocardial infarction. Hypertension and diabetes are more common in women, and overweight and/or obesity, fatty diet and smoking are more common in men.	7
280	Wang Xiaowei	Diet & Health Care	2021	Retrospective Cohort S	120	Young patients with AMI show a male predominance, with obesity, smoking, and alcohol consumption being common risk factors. Coronary artery lesions are predominantly single-vessel disease, and the clinical presentation is characterized primarily by chest pain.	6
281	Zhang Jing	MMJC	2021	Retrospective Case-Co	104	Youth under 40 years old who had smoking history, high fat and low cellulose diet, family history of coronary heart disease, heavy work pressure and irregular life rhythm are prone acute myocardial infarction. Young patients with acute myocardial infarction have more severe coronary artery lesions, which needs to arouse widespread attention from the society	6

282	Zuo Huijuan	Chinese Circulation Journal	2021	Retrospective analysis	2866	Proportion of young adults with first AMI is significantly higher in males than in females. The presentation of AMI is more frequently STEMI, but prevalence of NSTEMI increases over time. Observed trends changes are associated with changes on the multiple metabolic risk factors and characteristic of coronary artery disease in young adults suffering from first AMI	7
283	Zhang Xuefeng	Journal of China Health Care & Nutrition	2021	Retrospective Case-Co	88	The premature MI group had higher rates of smoking, hyperlipidemia, and family history of premature CHD compared to the control group; Hypertension, diabetes, stroke, and anemia were lower than the control group; Single-vessel coronary lesions were more common than in the control group, with frequent involvement of the LAD; Differences existed between premature MI and non-premature MI regarding infarct-related arteries and the number of diseased vessels.	6
284	Li Ying	Medical Journal of Chinese People's Health	2021	Retrospective Case-Co	70	Alcohol consumption was an independent influencing factor for premature AMI in males (OR≈9.5); Diabetes and HDL-C ≤ 1.04 mmol/L were independent influencing factors for premature AMI in females; The composition of risk factors for premature AMI differed between genders.	5
285	Han Yongsheng	Anhui Medical Journal	2021	Retrospective Case-Co	480	The premature STEMI group had higher proportions of males, smoking rates, hyperlipidemia, and family history of CHD compared to the control group, and lower hypertension; TG, LDL-C, ApoA, and ApoB were higher, while HDL-C was lower; Single-vessel lesions were more common, and multi-vessel lesions were fewer; The rates of malignant arrhythmias and in-hospital mortality were lower than in the control group.	7

286	Su Shaohui	Chin J Evid Based Cardiovasc Med	2022	Retrospective Cohort S	817	oung patients with acute ST-segment elevation myocardial infarction (STEMI) are predominantly male migrants. They have a better clinical prognosis compared to middle-aged and elderly patients, with smoking and obesity being more common risk factors in this group	7
287	Zhang Haihua	Chinese Circulation Journal	2022	Retrospective Case-Co	593	Compared with young STEMI patients, those with NSTEMI presented with a higher burden of risk factors, more severe coronary artery lesions, and a greater number of implanted stents. Furthermore, LDL-C and obesity were identified as independent correlates of NSTEMI	7
288	Kong Xiangyun	J Med Imaging	2022	Prospective Cohort Stu	81	Smoking, obesity, low-density lipoprotein cholesterol (LDL-C), and lipoprotein-associated phospholipase A2 (Lp-PLA2) are the most significant risk factors for young patients with ST-segment elevation myocardial infarction (STEMI). Coronary artery lesions in this population are predominantly single-vessel disease, characterized mainly by lipid-rich plaques, with plaque rupture being a common occurrence.	7
289	Zhao Haifeng	MMJC	2022	Retrospective Cohort S	481	Young patients with acute ST-segment elevation myocardial infarction (STEMI) are predominantly male. Smoking, overweight, obesity, uric acid (UA), triglycerides (TG), and low-density lipoprotein cholesterol (LDL-C) are the major risk factors for this population.	7

290	Chen Shuo	Shanxi Med J	2022	Retrospective Case-Co	70	A family history, smoking history, hypertension, and elevated uric acid are high-risk factors for ST-segment elevation myocardial infarction (STEMI) in young individuals. Unhealthy lifestyles among young adults — characterized by high work pressure, a fast pace of life, excessive fatigue, smoking, alcohol consumption, and binge eating — can all lead to disturbances in the internal physiological environment. These disturbances may contribute to coronary atherosclerosis and increase the incidence of acute myocardial infarction (AMI)	6
291	Li Xiaorong	Tibet Science and Technology	2022	Cross-sectional Study	300	Traditional cardiovascular risk factors, hemoglobin concentrations, and lipid profiles vary across different age groups, genders, and ethnicities among STEMI patients in high-altitude regions. Smoking, alcohol consumption, and a family history of cardiovascular disease are more prevalent in young patients. Furthermore, female STEMI patients tend to have an older age at onset and a higher prevalence of hypercholesterolemia.	6
292	Li Qi	Journal of Clinical Medicine in Practice	2022	Retrospective Case-Co	101	Clinical monitoring should be strengthened for women of childbearing age who have risk factors for coronary heart disease (CHD). In particular, early screening and preventive interventions are recommended for those who are overweight, have elevated serum triglyceride (TG) levels, or have a history of hysterectomy and/or oophorectomy.	6
293	Chen Qingli	Chinese and Foreign Medical Research	2022	Retrospective Case-Co	400	, BMI, exercise condition, drinking index, smoking index, family history of premature coronary heart disease, diabetes mellitus, hypertension, hyperlipidemia, depression and anxiety were the influence factors of occurrence of acute myocardial infarction of middle-aged and young people	8

294	Feng Jianzhao	Modern Medicine and Health Research	2022	Retrospective Case-Cc	72	A family history of premature cardiovascular disease, hypertension, sleep duration of less than 6 hours per day, total cholesterol (TC) > 6.47 mmol/L, and triglycerides (TG) > 2.26 mmol/L are independent risk factors for acute myocardial infarction (AMI) in middle-aged and young adults. Clinicians can utilize these indicators to guide disease prevention and management.	7
295	Jing Gao	Frontiers in Endocrinology	2022	Retrospective Cohort S	772	In patients with PMI, 1) hyperglycemia usually indicates more severe lesions; 2) MS as a whole was an independent risk factor for MACE; 3) BMI ≥28.0 kg/m2 and hyperglycemia were associated with MACE.	8
296	Yang Lincheng	Frontiers in Genetics	2022	Retrospective Case-Cc	2499	The PCSK9 R93C variant was associated with significantly reduced risk of PMI in the Chinese Han population, and the model we developed performed well in predicting PMI risk in this Chinese Han population.	9
297	Li Aizhen	Chin J Lab Diagn	2022	Retrospective Case-Cc	154	Smoking, sleep deprivation, and a family history of premature coronary artery disease are independent risk factors for young AMI. Hypertension and diabetes mellitus are uncommon in this population, which predominantly presents with single-vessel coronary artery disease and preserved ejection fraction (EF).	6
298	Ou Qiaoyun	Journal of Clinical Internal Medicine	2022	Retrospective Case-Cc	196	The premature male STEMI group had higher proportions of diabetes, non-HDL-C, and non-HDL-C/HDL-C ratio compared to controls, and lower HDL-C; Elevated non-HDL-C/HDL-C ratio was an independent risk factor for premature male STEMI (OR=2.96, 95%CI: 1.82 – 4.83); Its predictive value was superior to that of hypertension and diabetes.	7

299	Zhang Xin	Journal of Nanjing Medical University (Natural Sciences)	2022	Retrospective Cohort S	166	NGAL, creatinine, time from AMI to IRA opening, reduced LVEF, and IRA being the left anterior descending artery were independent risk factors for postoperative MACE in premature STEMI; A nomogram model was constructed accordingly, with internal validation AUCs of 0.95 at 6 months, 0.94 at 12 months, and 0.87 at 24 months, demonstrating good predictive efficacy.	6
300	Lu Jingru	Chinese Journal of Health Engineering	2022	Retrospective Case-Co	189	The young group had a high proportion of males, smokers, and higher Killip classification; High LVEF, low Gensini score, and a high prevalence of single-vessel disease; High proportion of onset between 06:01-12:00; Male gender, smoking, family history, and metabolic risk factors were independent risk factors; Young AMI often occurs in the morning.	6
301	Li Yang	Cardiovascular Disease Electronic Journal of Integrated Traditional Chinese and Western Medicine	2022	Retrospective Case-Co	340	The majority of young AMI patients were male. Common risk factors included obesity and smoking. Most patients presented with single-vessel coronary artery disease, and the predominant clinical manifestation was chest pain	6
302	Liu Fang	Chinese Journal of Art	2022	Retrospective Case-Co	180	The proportions of smoking, dyslipidemia, and family history in young AMI patients were significantly higher than those in the middle-aged and elderly groups; Coronary lesions were mainly single-vessel disease, while the proportion of multi-vessel disease was high in the middle-aged and elderly groups; The left anterior descending artery was most commonly involved in the young group, while the right coronary artery had a higher involvement rate in the middle-aged and elderly groups.	6

303	Zhao Ting	Chin J Cardiovasc Med	2023	Retrospective Cohort S	3858	Young STEMI patients exhibit an increased proportion of metabolic risk factors and a higher rate of adherence to guideline-recommended standard therapies, while in-hospital mortality has not shown significant changes.	7
304	Li Ruirui	Chin J Lab Diagn	2023	Retrospective Case-Co	525	Independent risk factors for AMI in young adults include elevated levels of uric acid, triglycerides, and low-density lipoprotein cholesterol (LDL-C). These metabolic abnormalities may be associated with unhealthy lifestyle habits such as irregular eating, staying up late, and physical inactivity. Improving lifestyle, adopting a healthy diet, and increasing physical exercise can help reduce the incidence of AMI in this population to some extent.	7
305	Deng Yifan	Chinese General Practice	2023	Retrospective Case-Co	1201	Smoking, total cholesterol (TC) $\geq$ 5.17 mmol/L, remnant-like particle cholesterol (RLP-C), and the atherogenic index of plasma (AIP) are identified as independent risk factors, whereas HDL-C serves as a protective factor. Furthermore, RLP-C and AIP demonstrate certain predictive value for early-onset acute myocardial infarction (AMI).	7
306	Hong Jiayuan	JOURNAL OF GUANGDONG MEDICAL UNIVERSITY	2023	Prospective Cohort	447	The clinical characteristics and short-term prognosis of young AMI patients differ significantly from those of elderly AMI patients. Killip class $\geq$3 and fibrinogen are independent risk factors for MACE within 1 year after discharge in young AMI patients.	7

307	Zhao Wenxing	Chin J Emerg Resusc Disaster Med	2024	Cross-sectional Study	120	Among the young population in high-altitude regions, diabetes mellitus, a history of smoking, and elevated levels of total cholesterol (TC), low-density lipoprotein cholesterol (LDL-C), diastolic blood pressure (DBP), and high-sensitivity C-reactive protein (hs-CRP) are independent risk factors for ST-segment elevation myocardial infarction (STEMI). Therefore, implementing targeted preventive measures and strengthening the management of underlying diseases are of great significance in reducing the risk of STEMI.	4
308	Nie Pengfei	Clin Intern Med	2024	Retrospective Cohort S	520	Male sex, a history of alcohol consumption, diabetes mellitus, and dyslipidemia are high-risk factors for ST-segment elevation myocardial infarction (STEMI) in young individuals.	7
309	Wan Suxia	Clinical Journal of Chinese Medicine	2024	Retrospective Cohort S	384	The risk factors and coronary artery lesion characteristics of male AMI patients differ across age groups. Young males should improve their dietary habits and increase physical exercise. Furthermore, early follow-up and regular check-ups are recommended for males with a family history of coronary heart disease (CHD).	7
310	Wang Zhe	Knowledge of Cardiovascular Disease Prevention and Treatment	2024	Retrospective Case-Co	80	A history of hypertension, an LDL-C level $\geq$ 3.4 mmol/L, and the presence of anxiety are independent risk factors for acute myocardial infarction (AMI) in young patients. Anxiety may contribute to the onset of AMI in this population, potentially by influencing blood pressure.	7
311	Chen YongZeng	Journal of Cardiovascular&Pulmonary Diseases	2024	Retrospective Case-Co	734	The severity of coronary artery lesions in young female AMI patients worsens with an increasing number of risk factors. It is significantly positively correlated with diabetes mellitus, serum uric acid levels, smoking, and a family history of AMI. Therefore, active and effective control of AMI risk factors is of great significance for preventing the occurrence of AMI in young women.	7

312	Sun Yanzhuan	Molecular Cardiology Journal	2024	Retrospective Case-Co	320	The early-onset MI group had hypertension, diabetes, low BNP, but good TG, LVEF, and renal function; Increased platelet count, elevated TG, and decreased HDL-C were associated with early-onset MI; Prognosis was good at 1 year post-surgery but bleeding risk was high.	7
313	Bai Yu	Journal of Vascular and Endovascular Surgery	2025	Retrospective Cohort S	206	The clinical characteristics of young patients with acute myocardial infarction (AMI) differ from those of middle-aged and elderly patients. Multiple factors are associated with the occurrence of AMI in young individuals, including smoking, alcohol abuse, a family history of coronary heart disease, hyperlipidemia, and obesity.	7
314	Yang Wentao	Chinese Circulation Journal	2025	Cross-sectional Study	28070	AMI patients with FHpCHD exhibit a clustering of family unhealthy lifestyle and cardiovascular risk factors, with an earlier onset age and more severe coronary artery disease. FHpCHD is associated with AMI, independent of lifestyle and cardiovascular risk factors.	7
315	Li Ying	Clin J Med Officers	2025	Cross-sectional Study	4993	Young AMI patients exhibit elevated levels of triglycerides (TG), total cholesterol (TC), and low-density lipoprotein cholesterol (LDL-C), along with reduced high-density lipoprotein cholesterol (HDL-C) compared to middle-aged and elderly patients. Furthermore, lipid profiles vary significantly across different combinations of risk factors	7
316	Zhao Jinging	Chin J Evid Based Cardiovasc Med	2025	Cross-sectional Study	324	Significant disparities exist in risk factors and coronary angiographic characteristics among ACS patients of different ages. Young patients are predominantly associated with unhealthy lifestyles and multiple metabolic risk factors, whereas the elderly are more prone to multi-vessel disease and severe coronary stenosis, resulting in a poorer prognosis.	6

317	Xu Wei	Journal of Chronicology	2025	Retrospective Case-Co	487	Gender, smoking history, alcohol abuse, hypertension, family history of coronary heart disease, initial symptoms of AMI, arrhythmia, cardiac insufficiency, and total cholesterol were identified as the primary causes of acute myocardial infarction in the ≤40-year-old population of Liaocheng City	7

Abbreviations: AMI, acute myocardial infarction; NOS, Newcastle-Ottawa Scale; PCI, percutaneous coronary intervention.

Note: For studies published in Chinese journals without an official English title, the original Chinese title is retained to ensure accurate identification. Author
